# Micronutrient Improvement of Epithelial Barrier Function in Various Disease States: A Case for Adjuvant Therapy

**DOI:** 10.3390/ijms23062995

**Published:** 2022-03-10

**Authors:** Katherine M. DiGuilio, Elizabeth Rybakovsky, Reza Abdavies, Romy Chamoun, Colleen A. Flounders, Ariel Shepley-McTaggart, Ronald N. Harty, James M. Mullin

**Affiliations:** 1 Lankenau Institute for Medical Research, 100 Lancaster Avenue, Wynnewood, PA 19096, USA; diguiliok@mlhs.org (K.M.D.); scimecae@mlhs.org (E.R.); 2 Department of Biology, Temple University, 1900 North 12th Street, Philadelphia, PA 19122, USA; reza.abdavies@temple.edu; 3 Department of Medicine, Lankenau Medical Center, 100 Lancaster Avenue, Wynnewood, PA 19096, USA; chamounr@mlhs.org; 4 Department of Food and Nutrition Services, Pennsylvania Hospital, 800 Spruce Street, Philadelphia, PA 19107, USA; colleen.flounders@pennmedicine.upenn.edu; 5 Department of Pathobiology, School of Veterinary Medicine, University of Pennsylvania, 3800 Spruce St., Philadelphia, PA 19104, USA; arielsh@vet.upenn.edu (A.S.-M.); rharty@vet.upenn.edu (R.N.H.); 6 Division of Gastroenterology, Department of Medicine, Lankenau Medical Center, 100 Lancaster Avenue, Wynnewood, PA 19096, USA

**Keywords:** micronutrient, tight junction, claudin, zinc, Vitamin A, Vitamin D, barrier function, inflammation, sepsis, virus, COVID, critical care

## Abstract

The published literature makes a very strong case that a wide range of disease morbidity associates with and may in part be due to epithelial barrier leak. An equally large body of published literature substantiates that a diverse group of micronutrients can reduce barrier leak across a wide array of epithelial tissue types, stemming from both cell culture as well as animal and human tissue models. Conversely, micronutrient deficiencies can exacerbate both barrier leak and morbidity. Focusing on zinc, Vitamin A and Vitamin D, this review shows that at concentrations above RDA levels but well below toxicity limits, these micronutrients can induce cell- and tissue-specific molecular-level changes in tight junctional complexes (and by other mechanisms) that reduce barrier leak. An opportunity now exists in critical care—but also medical prophylactic and therapeutic care in general—to consider implementation of select micronutrients at elevated dosages as adjuvant therapeutics in a variety of disease management. This consideration is particularly pointed amidst the COVID-19 pandemic.

## 1. Introduction

If you were a patient in a hospital bed and your physician walks into your room and tells you, “I have a formulation that won’t cure you, but it will improve your condition by 15%”, would you say, “Only 15%? No thanks”. No matter how mild your overall condition, you would instead likely take that offer gladly. A marginal improvement of morbidity is something anyone who has been in a hospital bed would not scoff at. For relatively mild conditions it would be welcome, but for severe conditions such as certain infectious diseases where mortality is an issue, a 15% improvement could be lifesaving, enabling a patient to maintain their physiology while their immune system is still ramping up to deliver the decisive blow against a pathogen.

A very diverse group of micronutrients have just this capability. Here we highlight the ability of three of these compounds to improve epithelial barrier function, the compromise of which is pivotal in the etiology of an extremely wide range of disease states. Epithelial barrier leak is at the heart of a great deal of morbidity—and mortality—climaxing in multiorgan failure that is such a battleground in critical care medicine. This is not surprising given that we—and animals generally—are physiologically a series and parallel array of sacs and tubes in terms of our tissue and organ architecture. Anything that undermines that compartmentation is an intrinsic threat to us. A plethora of diseases and pathogens do just that. Research over the past 35 years has, however, increasingly shown that there are naturally occurring compounds that support our intrinsic compartmentation, and work to protect it when our homeostasis is threatened.

Even more remarkable is that these compounds are generally safe. In fact, the FDA applies the GRAS (“Generally Recognized as Safe”) status on many of them. Moreover, many are safe at levels above their RDA (Recommended Daily Allowance) level. Using zinc as an example, this micronutrient—the second most abundant transition metal in the body—has a typical dietary intake of 5–10 mg/day [1]. The blood level of zinc is typically 10 micromolar (µM) [2]. Raising this level to 50 µM activates or amplifies cellular signaling pathways that, among many other diverse actions, modify the tight junctional (TJ) complexes around epithelial cells in diverse epithelial tissues (these seals being the lynchpin to our tissue and organ level compartmentation). Based upon simple Michaelis–Menten kinetics, zinc at a 5 µM concentration may have only negligible affinity for a binding site on a random signaling intermediate protein, but zinc at 50 µM may exhibit significant binding (and protein activation). In humans, this increased plasma zinc concentration is safely achievable by increasing zinc intake to 50–60 mg/day, roughly 5–10 times our normal dietary intake. *What is thoroughly remarkable is that this elevated zinc level not only modifies the TJ complexes, but also improves them*. They are less leaky basally and they are less prone to leakiness in disease states such as inflammatory cascades [3,4,5]. We say “remarkable” because the TJ complex is indeed a complex array of over 26 different barrier proteins and 20 or more junctional-associated intracellular proteins [6,7]. To *improve* such a byzantine structure by modifying its individual components is a huge biologically impressive feat.

The very safety and natural occurrence (and affordability) of these dietary compounds is ironically the likely cause of their perceived banality in the realm of medical therapeutics. “How important can zinc be?”, is a common unspoken thought by many a researcher and physician. Added to that is the large amount of “bad science” and misconceptions surrounding micronutrients. However, their very safety (and banality and affordability) is key to their implementation—prophylactically and therapeutically—because of their above-mentioned typical GRAS status and their well-studied toxicity limits. Any de novo drug that targeted TJ complexes would have to clear many safety trials and regulatory hurdles that GRAS compounds can bypass. What is pivotal to recognize, however, is that these compounds are drugs when applied at levels above their RDA, although drugs that have been “vetted” by our very evolution, given that we evolved over millennia with them in our diet, and very possibly, at levels above current RDAs.

**Figure 1:**
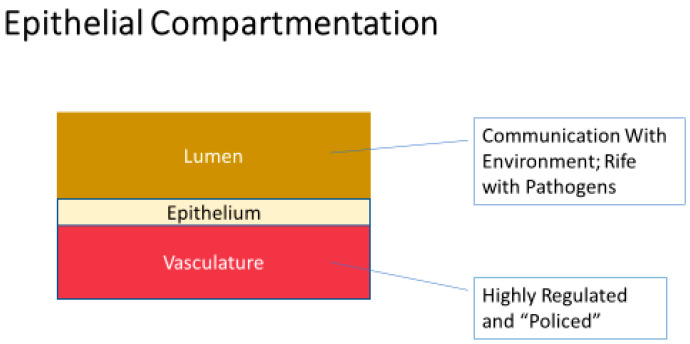
A very basic view of epithelial barrier function—separation of a multitude of different luminal compartments from the vasculature. Communication of these luminal compartments in many cases with the outside environment makes them rife with allergens and pathogens. The immune system, being situated primarily in the vascular compartment, places an enormous premium on barrier integrity segregating the immune system from its activators in these luminal compartments. Failure to separate can then lead to severe and/or chronic inflammation.

Many excellent reviews exist in the published literature dealing with the vital importance of epithelial and endothelial barriers, and the disease implications of their compromise [8,9,10,11,12]. Functional barriers are vital physiologically because they underwrite the very possibility of vectorial, unidirectional transepithelial/transendothelial transport; i.e., absorption and secretion at the tissue/organ level. However, in the case of epithelial barriers, they are also essential because they invariably sequester an immunologically “nasty” luminal (apical) compartment (often communicating with the outside environment) from the pristine systemic bloodstream (basal-lateral) compartment on the opposite side of the barrier. Those two worlds “mixing” in an unregulated manner is the genesis of a great deal of morbidity, and not simply in infectious disease. (Figure 1.)

In its first section (below), our review initially points out a wide variety of diseases that have barrier compromise at or near their core. We then pivot in the second section to illustrate instances where a micronutrient deficiency can induce barrier leak. This is followed in the third section by evidence where micronutrient *supplementation* (i.e., above normal dietary levels) induces barrier improvement and/or protection. In the final sections we discuss the application of these broad findings to actual clinical medicine.

Writing this review in the time of the SARS-CoV-2 pandemic, it is worth highlighting another very recent review that emphasizes the role in this pandemic of one particular micronutrient deficiency—zinc—along with zinc’s role in epithelial barrier function, and zinc’s ability to provide a cost-effective and easily applied remedy to decrease the number of COVID cases, and the severity of these cases, globally [13]. Our current review deals with both deficiency and supplementation of three micronutrients—zinc, Vitamin A and Vitamin D—describing the significance of each in the fight against COVID, as well as other diseases involving barrier compromise.

## 2. Is Epithelial Barrier Compromise a Common Occurrence in Disease?

### 2.1. Systemic Inflammation

There may be no better-described biological effector protein associated with transepithelial barrier leak than the proinflammatory cytokine. There is an enormous basic research literature describing the TJ-compromising activity of a variety of proinflammatory cytokines—Tumor Necrosis Factor-α, Inerleukin-1-β, Interleukin-6, Interferon-ɤ, and others—on a wide array of epithelial cell culture models [14,15,16,17,18,19,20]. There is similarly a very large, clinical literature describing how elevated systemic and tissue levels of proinflammatory cytokines associate with barrier leak in vivo. Nowhere is this situation more dramatically exhibited than in sepsis and its attendant multi-organ failure (MOF).

In any survey of the sepsis/MOF/cytokine published literature, the research conducted by Mitchell Fink’s group features prominently. Focusing mostly on the intestinal epithelial barrier, Fink’s group early on highlighted the interplay between sepsis/MOF/proinflammatory cytokines and barrier leak [21,22]. Animal studies have shown that the association holds for much more than simply the intestinal barrier. Huang and Gu [23,24] offer examples of blood–brain barrier (BBB) compromise. Gonzales [25] address the pulmonary barrier, both endothelial as well as epithelial. Rodrigues and Granger [26] deal more specifically with endothelial barrier compromise by cytokines and other soluble mediators in blood, as well as oxidation and reactive oxygen species. Mariano [27] focus on renal barrier function in this context and highlight how severely burned patients fit very well into this overall scenario of elevated cytokines, MOF and barrier leak.

An interesting clinical observation that links the phenomena of barrier leak, sepsis and MOF is that circulating levels of the epithelial and endothelial TJ-associated protein, ZO-1, show elevation in the blood stream of sepsis patients. Moreover, the degree of elevation correlates with APACHE II scores and SOFA scores and is statistically higher in the blood of sepsis patients who would go on to fail to recover from their condition [28,29].

### 2.2. Inflammatory Bowel Disease

There is absolutely no controversy over the fact that both CD and UC present with gastrointestinal barrier defects, i.e., transmucosal leak, although the type of leak may vary in the two diseases. Mankertz and Schulzke [30] characterize CD as being associated with TJ strand breaks, discontinuities and altered TJ proteins, whereas UC associates with upregulated epithelial apoptosis and micro erosions or “holes.” Perhaps no other disease is so associated with epithelial barrier leak, attested to by the very large number of published reviews dealing with the association of these two separate diseases with transepithelial leak.

Many of these reviews discuss the prevailing conundrum in IBD—which comes first, the epithelial barrier leak or other disease manifestations, most notably inflammation, that can generate such a leak. It is not an easily answered question because whereas inflammation—and specifically proinflammatory cytokines—can generate barrier leak, the leak itself can generate inflammation by virtue of allowing luminal antigens (and pathogens) to cross into the subepithelial compartment of the tissue. The various reviews invariably lean toward one mechanism or the other. It is quite a conundrum because no one disputes that leak can generate inflammation nor that inflammation can generate leak. Fakhoury [31] is an example of an IBD review that weighs both possibilities, and then discusses the implications for treatment options.

Among the earliest reviews on this topic is a landmark. Hollander [32] proposed that “increased intestinal permeability could allow the penetration of antigenic or infectious agents into the intestinal wall and thus start the process which in susceptible individuals culminates in Crohn’s disease”. He cites structurally abnormal tight junctions in even non-inflamed tissue from CD patients, as well as functional permeability defects (leak) in asymptomatic first-degree relatives of CD patients, as the basis for barrier leak being primary. Schulzke [33] present a modern twist to the thesis by delineating the two types of IBD leak described above and invoking the penetration of luminal antigens through such leak, as a major driver of tissue inflammation. Specific components of this inflammation, such as Interleukin-13, which they hold to be a major player in UC, in turn drive further leak through TJ alteration and induction of apoptotic cell death. Luettig [34] describe how claudin-2 is upregulated in IBD in both small and large intestine, resulting in TJ strand discontinuities that allow for transepithelial permeation of luminal antigens (and ensuing inflammation). Another example of reviews that lean toward barrier leak being primary is Teshima [35], who point to a genetic basis for such leak, namely, mutation in the NOD2 gene, also citing studies showing that rodent colitis models indicate barrier leak preceding inflammation. Takeuchi [36] had also explored a genetic base for IBD leak but point out that the leak observed in asymptomatic first-degree relatives does not display classical inheritance patterns.

Reviews leaning toward a primary role for inflammation (generating leak) in IBD are exemplified by Luissint [37] who discuss the barrier being affected by the inflammatory microenvironment and highlight particularly the role of leukocyte–epithelial interactions. This theme was explored earlier in Bruewer [38], who highlighted the role of Interferon-ɤ and TNF-α. Shen and Turner [39] describe the importance of mediation of effects by inflammatory proteins via the actin cytoskeleton and especially Myosin Light Chain Kinase. Odenwald and Turner [40] point out that animal studies show that barrier compromise is insufficient to generate IBD and that clinical trials focused on barrier improvement have not shown efficacy in IBD generally.

Zhu and Landy [41,42] offer a more general, neutral review regarding IBD and leak that focuses on claudin dysregulation and expand the conversation to epithelial-to-mesenchymal transition (EMT) of the tissue, along with its increased cancer risk. Fries et al. [43] in their review highlight the potential direct causes of IBD in the context of treatment options. The review by Larabi [44] focuses on less considered but highly germane topics such as the role of altered microflora and of dysregulated epithelial autophagy in IBD and its attendant barrier leak.

Regardless of whichever is the primary driver responsible for IBD—leak or inflammation—it is beyond question that barrier leak is an intrinsic component of IBD etiology—both UC and CD—though the type of leak involved in each may be different. Amid the “vicious circle” scenario, which seems well supported in the IBD published literature, all of the above reviews would seem to support a contention that amelioration of the leak would help the clinical situation.

Specific original research publications touch on individual aspects of the barrier leak and inflammation themes of IBD, but all appear to support the existence of transepithelial leak in the disease. Marin [45] observed varying degrees of TJ fragmentation in terminal ileal tissue of CD patients. Transmural electrical resistance was found to be 50% lower in inflamed sigmoid colon tissue from UC patients [46]. This was accompanied by an impaired TJ structure, namely, a decrease in strand count. Gitter [47] documented an increase in transepithelial conductivity in both mild and moderate-to severely inflamed UC sigmoid colon tissue. Foci of epithelial apoptosis contributed to leak in mild inflammation where epithelium appeared largely intact. In higher degrees of inflammation, mucosal erosions were major contributors to leak.

Numerous studies have investigated in more detail the changes to TJ proteins in IBD patients. An increase in claudin-2, as well as a downregulation of claudins -4 and -7 were observed in rectal epithelial mucosa biopsies from active UC patients [48]. Sigmoid colon biopsies taken from active CD patients exhibited decreased barrier function—as evidenced by a drop in epithelial resistance. Reduced and discontinuous TJ strands were seen in electron microscopy, leading to analysis of specific TJ proteins. There was an upregulation of claudin-2, whereas occludin, claudins -5 and -8 were downregulated [49]. Evaluating biopsies from active CD and UC patients, Das [50] observed an increase in claudin-2, in addition to reduced expression of ZO-1 and claudin-4. Lameris [51] saw an increase in claudin-12 in CD ileum as well as a downregulation of claudin-2 in sigmoid colon. They elaborate on various changes in claudin expression in UC and CD that are dependent on the intestinal segment or degree of inflammation.

Several rodent models of IBD have been utilized. Xu [52] observed significantly lower TER as well as an increase in claudins -1 and -2 in a dextran sodium sulfate (DSS) rat colitis model at days 7, 14, and 21. In a TNBS-induced mouse model of colitis, expression of claudin-18 was shown to be upregulated, a finding also seen in patients with UC [53]. In a DSS mouse model, Eraković Haber [54] noted a decrease in claudin-1 and claudin-3 in surface epithelium, and decreased claudin-8 in upper regions of crypts—a similar pattern to what was observed in UC patient biopsies.

The potential drivers of leak in IBD are the subject of many published studies. Serum zonulin, a biomarker of intestinal permeability, was found to be higher in IBD patients [55]. IL-10-deficient mice (a Crohn’s animal model) treated with the zonulin inhibitor, AT-1001, exhibited less small intestinal leak, resulting in attenuation of colitis, implicating leak as a contributing factor of disease induction [56]. In the SAMP mouse model of chronic ileitis, Olson [57] detected increased claudin-2 mRNA in the ileum and decreased occludin mRNA in the ileum and colon, additionally noting that increased permeability occurred prior to development of inflammation. Similarly, in DSS-induced colitis in mice, Poritz [58] measured an increased intestinal permeability accompanied by a loss of ZO-1 and increased claudin-1. Given an early loss of ZO-1 prior to significant onset of inflammation, they suggested that in this model it was possible for TJ abnormalities to precede inflammation. In a succeeding experiment, Poritz [59] first exposed the IEC-18 cell culture model to TNF-α, resulting in decreased TER, decreased occludin and increased claudin-1. Then, using IBD patient tissue, they demonstrated a pattern of increased claudin/occludin ratio. This was appreciated only in grossly diseased UC tissue; however, the ratio was elevated regardless of inflammation in CD. Therefore, they proposed that fundamental TJ alterations occur in CD. Another example of this occurred in IBD colorectal mucosal tissue, where there was a decrease in ZO-1, claudin-1, and occludin in areas of active inflammation with transmigrating PMNs. However, in areas with *only* mild active inflammation, occludin was still diminished [60].

Impaired barrier function also has implications in the clinical course of IBD. Söderholm [61] saw a larger increase in mucosal permeability in the distal ileum of CD patients in response to sodium caprate exposure as compared to experimental controls, suggesting TJs in CD are more susceptible to harmful luminal contents, thus implicating the influence of luminal “environmental” factors. This genetic and environmental interaction is supported in a prior study assessing L/M ratio patterns in CD patients, relatives and spouses after acetylsalicylic acid ingestion. In CD patients in clinical remission, an increased L/M ratio correlated with the probability of relapse [62,63,64]. Impaired intestinal permeability, as measured endoscopically by confocal microscopy and fluorescein leak, correlated with continued bowel symptoms in IBD even with endoscopic evidence of mucosal healing [65].

Additional evidence for genetic factor involvement in IBD also supports a causal role for barrier defects in IBD. Hollander [66] identified a two-fold increase in permeability (using polyethylene glycol maker) in CD patients and their healthy first-degree relatives. Increased intestinal permeability in healthy relatives of CD patients was replicated in other studies using the L/M ratio [67,68,69]. Peeters [69] also observed an increase in the L/M ratio in healthy spouses, pointing to environmental as well as genetic factors. Prospective studies following relatives of CD patients found a temporal correlation between increased intestinal permeability and the development of CD [70,71]. A number of studies have identified potential genes associated with increased IBD risk [72,73]. For example, mutations in the NOD2 (CARD15) gene have been linked with an increased susceptibility to IBD [74]. Furthermore, Buhner [75] reported that increased intestinal permeability in CD and their first-degree relatives (measured by L/M ratio) was associated with a CARD15 3020insC mutation.

Contrasting original research literature focuses on the primary role of inflammation and how inflammatory mediators perpetuate impaired barrier function and TJ complex abnormalities. For example, Gassler [76] report a downregulation of occludin in actively inflamed IBD tissue, inferring that alterations to the TJ complex are more likely a consequence of a primary inflammatory process. Several proinflammatory mediators, as well as potential molecular pathways have been investigated. In TNF-α-exposed Caco-2 cells, Ma [77] observed a disruption of ZO-1, as well as increased permeability—potentially mediated through NF-kappa B activation. Moreover, TNF-α decreased paracellular resistance of HT-29/B6 cells and increased claudin-2, which was thought to be mediated by the PI3K pathway [78]. Lamina propria mononuclear cells from UC patients exhibit increased production of IL-13. Exposure of HT-29/B6 cell layers to IL-13 results in decreased transepithelial resistance, a 3-fold increase in mannitol flux, a 1.2-fold increase in PEG flux and an increase in claudin-2 expression [79]. MLC phosphorylation has been shown to be significantly increased in biopsies of active IBD [80]. TNF-α and IFN used in combination decrease TER and increase MLC phosphorylation in Caco-2 and T_84_ cell layers [81,82]. This was accompanied by a reduction in occludin, claudins -1, -2 and -4 in T_84_ cells; however, claudin-1 increased slightly in the Caco-2 cell layers [82]. Prasad [83] also saw increased permeability in T_84_ cell layers exposed to TNF-α, IFN-ɤ or IL-13. IFN-ɤ and TNF-α treatment resulted in decreased claudin-3 and redistribution of claudin-4. An upregulation of claudin-2 was seen in T_84_ cell layers exposed to IL-13. They achieved this same pattern of results in sigmoid colon biopsies from IBD patients—a marked increase in claudin-2 with a concurrent decrease in claudins -3 and -4. Heller [79] also saw this increased claudin-2 expression in specimens from UC patients.

Also supporting a primary role for inflammation, excessive reactive oxygen species are known to promote intestinal inflammation (and barrier compromise) in IBD [84]. Microbiota have been found to contribute to this chronic inflammation. Bacteria producing hydrogen peroxide may play a role in increasing inflammation [85]. Firmicutes and Enterobacteriaceae were found to be in higher abundance in samples from IBD patients, likely contributing to oxidative stress pathways [86].

In summary, although the issue of barrier leak or inflammation being the primary cause in IBD is not settled, the reality of barrier leak in both CD and UC seems an established fact. Given the magnitude of the published literature surrounding it, IBD is actually the foremost example of barrier compromise being an integral element in a disease. The exact types of transepithelial leak have moreover been determined for the two diseases. With its well-developed human cell culture models and animal models, but also the ready availability of human tissue samples through colonoscopy procedures, IBD will be at the forefront of research involving epithelial barrier leak in disease.

### 2.3. Cancer

An often-overlooked property of lethal cancers is that approximately 95% are epithelial in origin [87]. The paradigm properties of epithelia—their intrinsic polarity and their ability to form TJ seals/functional barriers—thus become core issues in cancer biology. Effects of cancer on epithelial polarity are nicely summarized in Saito [88] as well as Hinck and Nathke [89]. The changed barrier aspect of neoplasia is our concentration here.

Basic research on epithelial cell culture models going back many years has shown that transformation/neoplasia of epithelial cells proceeds with, among other changes, alterations of their TJ complexes [90,91]. Evidence has been shown in hepatocellular, mammary and colon adenocarcinoma [92,93,94,95,96]. Soler [96] in fact purported to show a progression regarding increasing TJ leak, ranging from normal colon mucosa to junctions of hyperplastic and adenomatous polyps to actual colon adenocarcinoma.

Certain TJ-associated proteins have been found to function as tumor suppressor proteins [97,98,99,100]. In addition, the tumor promoter class of chemicals, such as TPA (12-O-tetradecanoylphorbol 13-acetate)—intricately associated with the overall chemical carcinogenesis process—has been observed to induce TJ leakiness through their activation of Protein Kinase C isoforms [101,102,103,104]. The potent oncoprotein, TGF-β, has been shown to disrupt epithelial TJs, as does activation of the erb-B2 receptor [105,106].

Just as beta-catenin is not simply a structural protein, there is a steadily growing awareness that certain TJ barrier proteins and TJ-associated proteins possess roles that extend beyond merely permeability and barrier function, a realization helped along by changes in these proteins in cancer. It is true that the leak arising from the dysregulation of a TJ barrier can lead to altered receptor-mediated signaling by virtue of altered tissue-level compartmentation of growth factors and other ligands [107]. However, there is a growing awareness that TJ and TJ-associated proteins can critically function as signal transduction mediators in their own right. This has been well described for ZO-1 and ZONAB [108,109]. It is also implicit in the existence and importance of “cytoplasmic” pools of these proteins that are quite distinct from a cytoskeletal association [110]. This ability of TJ proteins to function as “signaling intermediates” has been the subject of many recent reviews [111,112,113,114,115].

### 2.4. Celiac Disease

Celiac Disease is an intestinal autoimmune disease triggered by the presence of a component of the dietary gluten protein, gliadin, in the GI lumen of susceptible individuals, and those presenting the human leukocyte antigen (HLA)-DQ2 and/or (HLA)-DQ8 haplotypes. These haplotypes enable presentation of immunogenic gliadin peptides to gluten-specific CD4+ T-cells in the lamina propria. This leads to an immune cascade in the lamina propria which includes a proinflammatory cytokine upregulation. This inflammatory reaction then induces mucosal changes such as villous atrophy, but also impairment of barrier function [116]. There have been numerous excellent reviews identifying and describing epithelial barrier compromise in Celiac Disease [116,117,118,119,120,121,122,123,124].

Although an intestinal barrier impairment in Celiac Disease is well established, it is less clear whether the barrier impairment has a primary role in the disease or is only the secondary result of an inflammatory cascade in the mucosa, a situation very like that in IBD as described above. There are 39 gene loci showing variation in Celiac Disease patients, at least four of which are known to play roles in cell–cell adhesion (*LPP*, *C1orf106*, *PTPRK* and *PARD3*), one piece of evidence suggesting a primary barrier defect may exist in Celiac Disease [125,126]. The *PARD3* gene may have a role in epithelial polarity and TJ regulation as well [121]. Jauregi-Miguel [127] reported altered expression of the genes *CLDN2*, *PARD6A*, *ZAK*, *SYMPK*, *MYH14*, *ACTB*, *MAGI1*, *TJP1* (ZO-1) and *PPP2R3A*, which could further support a primary role for barrier compromise.

Whether the barrier compromise is primary or secondary in Celiac Disease, structurally altered TJ complexes and changes in TJ claudin composition are well documented. Evidence exists of a decreased TJ strand number and increased strand discontinuities in duodenal biopsies in Celiac Disease [128,129]. In addition, abundant functional evidence exists for an altered barrier, ranging from increased lactulose/mannitol (L/M) ratios in urine [124] to decreased transepithelial electrical resistance in ex vivo studies of intestinal biopsies in Ussing chambers [130]. In another parallel to the situation in IBD, first-degree relatives of Celiac Disease patients—themselves without manifestations of active disease—showed increase functional intestinal barrier leak as evidenced in higher L/M ratios [131]. These first-degree asymptomatic relatives also showed decreased levels of ZO-1 and occludin in their intestinal biopsies, as well as TJ ultrastructural abnormalities. Similarly, although TJ length was not altered in ultrastructural analyses of intestinal biopsies of very early stage Celiac Disease patients (presence of Celiac Disease-specific autoantibodies in sera but normal histology of the small intestine with increased number of IEL but no other features of inflammation), alterations in the desmosomes and dilated intercellular spaces were observed [132]. Downregulation of occludin in Celiac Disease is particularly noteworthy because it has been associated with increased macromolecule leak across Caco-2 intestinal barriers [133], a situation that would be permissive for paracellular leak of gliadin peptide from the lumen into the lamina propria. Altered ZO-1 localization, phosphorylation and expression is likewise commonly reported in active Celiac Disease [134,135]. Barmeyer [121] demonstrated lower claudin-5 levels in active Celiac Disease. Goswami [136] reported reduced expression of claudins -3 and -4, with overexpression of claudin-2. Szakál [137] also reported overexpression of claudin-2.

This controversy of whether a barrier leak in Celiac Disease is primary or secondary, may hinge on how and when gliadin and its component immunogenic peptides are able to move across the intestinal barrier. One can ask how do gliadin and/or gliadin peptides enter the lamina propria immunogenically intact in the first place? Hollon and Lammers [138,139] showed using human intestinal biopsies that incubation of the tissue with gliadin could compromise barrier function, establishing that gliadin peptide access to the lamina propria can *induce* leak. Sander [140] had earlier shown this using Caco-2 cell layers—an induced leak accompanied by changes in TJ barrier proteins. However, Ménard [141] had shown that *transcellular* transport of gliadin peptides is increased in Celiac Disease patients, a phenomenon that could give rise to leak without a primary paracellular defect.

There is also abundant literature showing increased tissue and serum levels of the intestinal protein, zonulin, in active Celiac Disease [131,142]. Alterations in zonulin signaling within intestinal epithelia may be pivotal in the observed barrier dysfunction in Celiac Disease. Zonulin has been a key target in potential Celiac Disease therapy using the compound, Larazotide, a compound that represents one of the first interventional attempts at therapeutically redressing a barrier compromise [143].

### 2.5. Infectious Disease

#### 2.5.1. Gastrointestinal Bacteria

The majority of research studies addressing the topic of bacterial effects on epithelial barriers comes from gastrointestinal (GI) epithelial models and GI pathogens. Moreover, the thrust of much of the research is directed at a single question—is infectious diarrhea due to paracellular barrier leak or is it arising from a transcellular secretory mechanism, or both? The answer is seemingly microbe specific.

Campylobacter-induced enteritis is a highly common food-borne source of diarrhea in humans. Work with colon biopsies and human GI epithelial cell culture models has shown disruption of TJ complexes with localization changes in claudins -1, -3, -4, -5 and -8 as well as induced focal cellular apoptosis and detachment, giving rise to two distinct paracellular leaks with the macromolecule leak increased as well as decreased TER. In addition, the ENaC sodium channel is affected, indicating that both transcellular and paracellular pathways are involved in Campylobacter-driven diarrhea [144,145,146]. This specific source of diarrhea and the mechanisms responsible for it are reviewed in Lobo de Sa [147], including a consideration of the role played by elevated mucosal proinflammatory cytokine levels.

*Acrobacter butzleri* infection of the HT-29/B6 cell layers was shown to both decrease TER and increase transepithelial fluorescein-dextran (4 kDa) diffusion, along with decreased expression of claudins -1, -5 and -8, as well as mislocalization of claudins -1 and -8. Focal epithelial apoptosis was also increased [148,149].

*Yersinia enterocolitica* also decreased TER dramatically in HT-29/B6 cell layers, and was accompanied by increased transepithelial diffusion of ^14^C-D-mannitol and fluorescein. Decreased expression of claudins -2, -3, -8 and -10 accompanied these changes as well as focal necrosis (increased LDH release) with cellular redistribution of claudins -3, -4 and -8 off from the TJ complex in the specific regions of focal leak, all providing evidence for paracellular leak-driven diarrhea [150].

The aerolysin toxin produced by *Aeromanas hydrophila* both induced chloride secretion as well as increased FITC-dextran (4 kDa) transepithelial diffusion across HT-29/B6 cell layers, processes seemingly transduced by altered myosin light chain kinase and intracellular Ca^++^ signaling [151]. This provides evidence for both secretory and leak-flux diarrhea for this infectious microbe.

Alpha-hemolysin-expressing *E. coli* induced barrier compromise of porcine colon tissue as exhibited by reduced TER. This associated with both focal leak formation (cell extrusions) as well as modification of the junctional barrier proteins, claudins -4 and -5 [152]. Similar results were seen in mouse colon and in *E. coli*—exposed HT-29/B6 cell layers [153].

*Staphylococcus aureus* enterotoxin B induced barrier leak across rat jejunum as exemplified by decreased TER and increased horseradish peroxidase transepithelial permeation. The effect was attributed to TJ barrier compromise with reduced TJ protein expression [154].

Exposure of Group A Streptococcus to Caco-2 intestinal cell layers dramatically reduced TER and reduced expression of occludin and tricellulin. Plasminogen was found to be a molecular bridge between bacterial surface enolase and the cell layer. More specifically, site-directed mutagenesis experiments showed lysine residues on the extracellular loop of tricellulin to be the cellular point of contact [155].

Curiously, exposure of T_84_ cell layers to *Neisseria meningitides* resulted in both decreased TER and increased ^3^H-inulin leak but was thought to be without accompanying TJ barrier change, as evidenced by no change in ZO-1 localization [156].

Perhaps the best studied of the GI pathogenic bacteria with regard to targeting the TJ is *Clostridium perfringens*, and specifically its enterotoxin. Work from notably the McClane group has shown the highly precise targeting of the TJ complex by this pathogen. Specifically, the C-terminal portion of the enterotoxin has specific interactions with the ECL-1 and ECL-2 extracellular loops of specific claudins [157]. Paracellular pores were formed in the TJ complex in Caco-2 cell layers by aggregate interactions of the enterotoxin with specific claudins [158]. Perturbation of the TJ complex by prior treatment with calcium chelators or even TNF-α enhanced this pore formation [159]. Claudin-4 is held to be one of the receptive claudins in the TJ complex [160].

#### 2.5.2. Non-Gastrointestinal Bacteria

For evidence of non-GI epithelial barrier compromise by bacterial pathogens, nasal epithelia have been a frequent model. In an early study on the process of transepithelial invasion of nasopharyngeal epithelial organ cultures, *Hemophilus influenza* type b was observed to induce TJ compromise followed by paracellular migration of bacteria. However, *Neisseria meningitidis* was observed to transmigrate transcellularly with intact TJ complexes in electron micrographs [161]. *H. influenzae* was later found to induce downregulation of claudins -7 and -10 by a TLR- and SNAIL-dependent mechanism in the nasal epithelium, a phenomenon also observed with exposure to *Streptococcus pneumoniae* [162]. *Staphylococcus aureus* caused a decrease in electrical impedance and a mislocalization of ZO-1 in the nasal epithelium, with actual TJ discontinuity in electron micrographs [163]. Martens [164] observed decreased ZO-1 and occludin expression with *Staphylococcus aureus* exposure to nasal epithelium, and also evidenced a TLR-dependent mechanism.

In human keratinocyte cell layers, *Staphylococcus aureus* downregulated not only the TJ proteins but also atypical Protein Kinase C, coincident with reduced transepithelial TER. Interestingly, *Staphyloccoccus epidermidis* had a much weaker effect [165]. In uterine epithelial cell layers, *Neisseria gonorrhea* curiously had no effect on either occludin or ZO-1 expression or localization, even though there was a dramatic effect on E-cadherin localization [166]. In brain microvascular endothelial cell layers, *Staphyloccocus aureus* exposure induced barrier compromise as well as reduced expression of claudin-5 and ZO-1 [167]. In capillary endothelia exposure to anthrax toxin also induced barrier compromise (increased leak of 3 kDa dextran) while also inhibiting p38 signaling [168]. In a different vascular endothelial cell culture model, *E. coli* induced dramatic transendothelial leak to 40 kDa dextran accompanied by significant elevation of intracellular calcium [169].

A noteworthy and counterintuitive study comes from work on human gingival epithelial cell layers. When these cell layers were exposed to the oral *commensal* bacteria, *Streptococcus gordonii*, upregulation of ZO-1, ZO-2, JAM-A and occludin occurred, along with *improved* barrier function as revealed by reduced leak of fluorescent-labeled dextran, highlighting the very finely tuned nature of microbial regulation of our epithelial and endothelial barriers [170].

Additional excellent and more extensive reviews can be found on barrier compromise by various bacterial pathogens in a variety of epithelial barrier cell types [159,171,172,173].

#### 2.5.3. Viral Pathogens

Viruses have evolved to utilize several mechanisms for efficient entry into host cells and subsequent spread across host barriers. Depending on the viral family, the mechanism differs, but the end goal remains the same: maximize spread and infectivity into host tissues. The coxsackievirus and adenovirus receptor (CAR) was the first TJ protein to be identified as a viral receptor for adenoviruses and coxsackie B viruses (reviewed by Freimuth [174]). Since then, many studies have sought to identify how other viruses engage with the TJ complex during infection [175]. Here, we highlight key findings from studies on how rotaviruses, flaviviruses, influenza viruses and coronaviruses interact with host TJ proteins and epithelial barriers during infection. One is left to conclude that virus evolution has focused intensely and elegantly on disruption of the endothelial and epithelial barriers to enhance virus spread through an organism.

##### Rotaviruses

Rotaviruses (RRV) are nonenveloped double-stranded RNA viruses that can cause severe gastroenteritis in infants worldwide. Diarrhea being a major manifestation of RRV infection, the human GI Caco-2 cell model has been used to study the effect of RRV infection on epithelial barrier function. Key studies demonstrated that expression of RRV protein, VP8, led to disorganization of occludin in TJs [176], and additional studies revealed that VP8 could also alter the cellular location of TJ proteins ZO-1 and claudin-3 [177]. These studies concluded that expression of VP8 allows the virus to generate ‘leaky’ TJs that permits RRV in an apical/luminal compartment to permeate paracellularly to its host receptor proteins, the integrins, located on the basal-lateral cell surface, thereby allowing efficient viral entry into the cell. Although RRV infects mature enterocytes in the intestinal epithelium, some studies utilized cultures of MA104 (epithelial monkey kidney cells), since these cells are highly permissive for virus replication. These studies showed that TJ proteins JAM-A, ZO-1 and occludin were required for RRV entry into this cell type [178], and that the RRV spike protein engaged JAM-A as a coreceptor for entry. Lastly, in addition to roles for VP8 and Spike proteins in TJ interactions, the RRV NSP5 protein, a secreted enterotoxin, was shown to induce leak across MDCK-1 renal epithelial cell layers [179]. These authors concluded that certain RRV viral proteins can function as complex entry machinery that results in multiple routes of cellular infection by first impairing normal function of TJs. Interestingly, low zinc serum levels have been associated with a higher risk of RRV infection, regardless of rotavirus vaccine status [180], while Vitamin D has been shown to alleviate RRV infection [181], indicating that micronutrient supplementation may be useful as a prophylactic treatment against RRV.

##### Flaviviruses

Many of the enveloped, positive-sense RNA viruses within the *Flavivirus* family are transmitted by arthropod vectors, such as the mosquito-borne pathogens, Zika virus (ZIKV), West Nile virus (WNV) and the four dengue virus serotypes (DENV1–DENV4). The severe form of the disease caused by DENV is referred to as dengue hemorrhagic fever (DHF), in which a drastic increase in endothelial permeability can lead to hypovolemic shock in patients. Viruses detected in brain microvascular endothelial cells led to the investigation of DENV infection in human dermal microvascular endothelial cells (HMEC-1). This showed actin cytoskeleton rearrangements and displacement of occludin from TJs in this cell type [182]. Further studies identified that the secreted form of the flaviviral protein, NS1, acts a pathogen-associated molecular pattern (PAMP) to induce pro-inflammatory cytokines that lead to increased vascular leak in vitro and in vivo [183,184]. DENV NS1 was also shown to directly alter endothelial barrier function by disruption of the endothelial glycocalyx-like layer in cultured human pulmonary microvascular endothelial cells (HPMEC) [185].

Interestingly, the NS1 protein is well conserved among flaviviruses. NS1 from DENV, ZIKV, WNV, Japanese encephalitis (JEV) and yellow fever viruses (YFV) was found to bind tissue-specific endothelial cells and alter transendothelial permeability based on the tropism of each virus [186]. In addition to NS1, the WNV capsid protein was shown to be sufficient to downregulate expression of the TJ protein, claudin-2, in the proximal tubules of the kidney in mice, while the TJ proteins, claudin-1 and JAM-1, were shown to be degraded in Caco-2, MDCK and HUVEC cell monolayers infected with WNV [187,188]. Similarly, in ZIKV-infected placentae, paracellular permeability was found to increase, with claudin-4 protein levels being reduced at the basal surface of the syncytiotrophoblast layer, suggesting that ZIKV alters the composition of placental TJs to enhance spread [189]. Since ZIKV infection causes microcephaly in infants, studies have also focused on ZIKV infection of primary human brain microvascular endothelial cells (BMECs). Although BMEC permeability was not affected by ZIKV infection, Leda et. al. showed that both occludin and claudin-5 levels were significantly downregulated during infection [190]. These studies show that regulation of TJ proteins and reduction in barrier function are strategies utilized by many members of the Flavivirus family.

This family also includes the hepaciviruses, such as the Hepatitis C virus (HCV) that causes viral hepatitis. TJ protein, claudin-1, was first shown to be necessary for cell culture-replicating HCV entry into human hepatoma cell lines, as well as retroviral particles pseudotyped with HCV E1 and E2 [191]. Later studies confirmed the role of claudin-1 in HCV entry and additionally found that claudins -6 and -9 also functioned well as cofactors for entry into CD81^+^ human endothelial cells but functioned poorly in hepatoma cells [192]. Additionally, HCV infection altered TJ function with changes in localization of occludin and claudin-1 in hepatoma Huh7 cells, with occludin being required for HCV pseudotyped particles entry [193,194]. In an elegant study from Baktash [195], single particle imaging then confirmed HCV accumulation colocalized with claudin1 and occludin at TJs, resulting in internalization of particles via clathrin-mediated endocytosis into a three-dimensional polarized hepatoma system [195]. Additionally, the role of micronutrients such as zinc, vitamin A and D in both acute and chronic infection of HCV is nicely summarized in a review by Gupta [196], who highlight that combined vitamin A and D deficiency was found to be a strong predictor of patients non-response to HCV antiviral therapy.

##### Influenza Viruses

Influenza viruses belong to the Orthomyxoviridae family and are classified into A, B and C types based on their core proteins. The Influenza A virus (IAV) is a respiratory pathogen that infects both humans and animals [197]. The IAV NS1 protein contains PDZ-binding motifs (PBMs) located at the C-terminal, consisting of four amino acid residues, which bind to cellular proteins that contain PDZ domains [198]. The avian strain, H5N1 NS1 protein contains a PBM with the sequence ESEV that was found to associate with PDZ proteins, MAGI-1, MAGI-2, MAGI-3, Scribble and Dlg1. Infection of MDCK cells with this strain showed a decrease in TJ integrity and an increase in paracellular permeability over the course of infection, while strains containing other PBM sequences, such as ESEA, did not display similar TJ disruption [198,199]. Further studies with H5N1 and H1N1 strains of IAV showed that infection of co-cultures of epithelial and endothelial cells as a model for the alveolar epithelial–endothelial barrier resulted in loss of the TJ protein, claudin-4, in only the epithelial NCl-H441 cells [200]. Interestingly, a meta-analysis of studies on the effects of Vitamin D and zinc on influenza infection concluded that both micronutrients reduced the duration of symptoms as compared to no supplementation [201].

##### Coronaviruses

Coronaviruses (CoV) are a family of viruses that infect a wide range of species and are associated with respiratory, GI and/or neurological diseases. Mouse hepatitis virus (MHV) infection causes primary hepatic, respiratory, or enteric disease, followed by neurological disorders in mice [202], and is often used as an animal model of CoV neuroinvasion. In a study by Bleau [203], a highly hepatotropic MHV3 strain increased blood–brain barrier permeability and lowered ZO-1, VE-cadherin and occludin, but not claudin-5 protein expression in murine BMEC cells [203]. Similarly, infection of porcine intestinal epithelial cells (IPEC-J2) with porcine epidemic diarrhea virus (PEDV) and transmissible gastroenteritis virus (TGEV) altered expression of ZO-1 and occludin, 1 h post infection, which correlated with increased permeability across the epithelial cell layer [204]. Further study of PEDV determined that occludin expression is increased but also displaced from the TJ complex during PEDV infection, with occludin possibly serving a scaffolding function after virus attachment to IPEC-J2 cells [205]. It should be noted that due to the prevalence of PEDV and TGEV in the pork industry, several studies have looked at the effect of Vitamin D and zinc supplementation on piglet disease progression [206,207,208,209,210], emphasizing the importance of translational medicine research into micronutrients on viral infection.

The 2020–2022 global virus pandemic was caused by the human CoV called Severe Acute Respiratory Syndrome-2 (SARS-CoV-2), named for its genetic similarity to the SARS-CoV-1 virus outbreak in 2002 [211]. Both SARS-CoV viruses target the epithelial cells of the respiratory tract, and in severe cases, patients develop diffuse alveolar damage and consolidation of the lungs, suggestive of a leaky pulmonary barrier [212,213] Teoh [214] showed that the small Envelope (E) protein from SARS-CoV-1 interacted with the TJ-associated protein, PALS1, via a PBM at its C-terminus. Ectopic expression of E redistributes PALS-1 away from the TJ complex in Vero E6 cells and delays formation of TJs [214]. Follow-up studies on the E protein from SARS-CoV-2 predict improved binding and increased affinity of the novel viral E protein to PALS-1, which may account for the enhanced infectivity and spread of SARS-CoV-2 [215,216,217]. Additionally, the PBM of the SARS-CoV-2 E protein has been shown to interact with the PDZ domain-containing TJ protein, ZO-1 [218]. This interaction is predicted to compromise epithelial barriers and cause TJ damage in cell layers, leading to enhanced virus spread and respiratory morbidity in patients.

Regardless of the role played by TJ modulation, micronutrients such as Vitamin D are thought to play a useful role in reducing COVID morbidity, as a timely example of micronutrient action on viral infection [219]. An inhibitory effect of Vitamin D on SARS-CoV-2 replication in fact has been advanced and ascribed to Vitamin D binding to the viral proteins, Mpro (main protease) and RdRP (RNA-dependent RNA polymerase) [220]. Binding of Vitamin D to the endoribonuclease, Nsp15, has also been reported [221]. Zinc, another micronutrient believed useful in reducing COVID severity, also binds to SARS-CoV-2 Mpro, an action that is potentiated by the micronutrient quercetin [222].

### 2.6. Diabetes

There is an enormous, published literature showing that increased leak across epithelial and endothelial barriers is an integral part of diabetes. In keeping with diabetes’ systemic presentation, the leak appears to encompass any endothelial or epithelial tissue that one can think of. The blood–brain barrier (BBB) and blood–retinal barrier (BRB) are two very prominently researched examples, but the phenomenon appears universal throughout the human body. The BRB leak is one of the oldest reported examples in the literature, with analyses of altered BRB TJs in diabetic rats by electron microscopy being one example [223]. The Antonetti group has published a great deal on the BRB phenomenon, early on reporting the decreased occludin content and increased paracellular leak that accompanies it [224]. Frey and Antonetti [225] have more recently reviewed the assembly of cellular and molecular mediators of this phenomenon. Klassen [226] reminds us that typical of the complexity of diabetes, however, it is not that simple, and dysregulated transcellular transport is at play here as well. Ocular pathology is not limited to the BRB either, as compromised barrier function—and altered TJ proteins—have been shown in mouse and human corneal models as well [227].

For the BBB, numerous reviews describing barrier compromise and TJ alteration exist, such as Banks [228] and Prasad [229], which moreover show that the theme of barrier compromise is associated with both type-1 and type-2 forms of the disease. As in other tissues, the TJ alteration induced by the diabetic state (decreased occludin, claudin-5 and ZO-1) may follow from an induced proinflammatory state in the barrier cells, as dysregulation of NF-kB, IL-1β, IL-6 and TNF-α have been observed [230,231].

The GI tract has been a focus of diabetes-associated barrier leak for two separate reasons. The first and simplest is that this is yet another barrier that manifests leak in diabetes. For example, in a mouse model a high-fat dietary-induced prediabetic state showed a significant reduction in claudins -1, -2 and -3, in ZO-1 in the duodenum and jejunum, but increased duodenal paracellular leak [232]. Streptozotocin-induced diabetes in rats also associated with decreased expression of several TJ proteins and increased barrier leak of ^14^C-sucrose [233]. In humans, obesity-linked type-2 diabetes occurred with a reduction in jejunal occludin and tricellulin as well as increased jejunal transepithelial permeability in a patient subset [234]. Horton [235] had earlier shown increased ^51^Cr-EDTA intestinal leak in type-2 diabetes. This being the GI tract, the discussion of diabetes-related barrier leak leads of course to consideration of diabetes-related change in the microbiome, and its effects on barrier function as well as a consideration of the effects of endotoxin leak across the leakier barrier [236]. This diabetes-related change in the GI microbiome is the second reason why the GI tract is a major focus of diabetes-induced change in epithelial tissues. The diabetes–GI barrier literature often points out that diabetes-associated GI leak often occurs in the context of increased inflammatory mediators in the leakier mucosal tissue [235,237].

In keeping with our earlier statement, that barrier leak in diabetes is systemic and across many tissue types, diabetes-associated hyposalivation was shown to associate with altered TJ protein expression in parotid glands [23]. In addition, gestational diabetes in a rat model induced increased leak across the placental barrier along with decreased ZO-1 and occludin expression [238].

### 2.7. Dust Mites

Although admittedly a bit eclectic, no better example exists than the dust mite of how diverse the biomedical examples are of disease morbidity involving compromised barrier function, and its associated rhinitis. Dust mite allergens have been found to comprise proteolytic enzymes in dust mite feces that are able to cleave nasal epithelial TJs (including the occludin protein itself), and thereby increase nonspecific transepithelial leak [239]. Specific cleavage sites on the occludin protein have been found [240]. Although such proteases have been linked to induction of apoptosis, the effect on TJs appears to be independent (although apoptosis induction may likewise also compromise barrier function) [241]. The Der p 1 antigen in dust mite feces has also been observed to decrease expression of the TJ component proteins, claudin-1 and JAM-A, in sino-nasal epithelia [242]. Using nasal epithelial cultures from controls vs. patients with dust mite-associated rhinitis, Steelant [243] also observed decreased occludin (as well as ZO-1) expression and impaired barrier function. The Der p 2 antigen was also observed to increase expression of claudin-2 [244]. The effect is not confined to the sino-nasal epithelium either. The Der p 1 antigen has been found in the human intestinal mucosa. In mouse and human studies, it was observed to associate with decreased GI expression of several TJ proteins and increased TNF-α, a study wherein the authors also speculate on a role in IBS [245]. Ma [246] interestingly observed that topical Vitamin D application in air–liquid interface cultures may alleviate dust mite-induced barrier compromise.

The dust mite studies are excellent examples yet again of how pathogen evolution appears to have placed a focus on our TJ seals. Dust mites moreover are only an example of the airway barrier compromising effect of a wide variety of allergens [247,248].

In summarizing this section on disease and barrier leak, one can very confidently state that barrier compromise appears to be an integral component of the etiology of an incredibly broad array of diseases. Inflammation is a typical common factor here, but the association of barrier leak with disease may be considerably more nuanced than simply deriving from inflammatory mediators.

## 3. Does Micronutrient Deficiency Lead to Barrier Compromise and Exacerbate Disease?

The published literature is replete with studies that have shown a variety of specific micronutrient deficiencies result in compromised epithelial barrier function across a variety of epithelial tissues with a variety of associated morbidities. The literature here is sufficiently large that to keep this review manageable, we are focusing on only three specific micronutrients: zinc, Vitamin A (retinoic acid, retinol, retinyl esters) and Vitamin D (calcitriol, cholecalciferol). These three micronutrients have the best developed body of evidence supporting a causal association between deficiency and impaired epithelial barriers.

### 3.1. Zinc Deficiency

An excellent review on the topic of zinc deficiency and disease is that by Prasad [249]. This review highlights zinc deficiency’s prevalence worldwide and points out that it is not simply associated with malnutrition but can occur with a high dietary phytate intake and can be common among the elderly even in developed countries. It clearly summarizes the association of zinc deficiency with diarrhea, especially infectious diarrhea in children. The link between deficiency, diarrhea and impaired GI barrier function is discussed in a review by Davidson [250].

The most abundant literature concerning zinc deficiency and impaired epithelial barrier function is in the GI tract. Using a human GI cell culture model (Caco-2), a zinc deficient culture medium (by zinc chelation) was found to decrease transepithelial electrical resistance (TER); decrease the overall amount of the TJ proteins, ZO-1 and occludin; decrease the phosphorylation state of occludin; and partially delocalize occludin and ZO-1 from each other and from the TJ band [251]. Although the focus with zinc in investigations with GI models typically focuses on TJs, Ranaldi [252] points out that the Caco-2 cell layers in a zinc-deficient state respond to TNF-α exposure with increased rates of cellular apoptosis in an NF-kB-mediated process that would also impair barrier function.

The evidence coming out of animal studies typically takes the form of zinc deficiency in concert with another physiological stressor. For example, dextran sodium sulfate-induced colitis in rats is exacerbated by a state of zinc deficiency in the animals, further compromising an already impaired GI barrier function [253]. Knockout mice defective in the zinc transport protein, ZIP14—a genetic manipulation that models a dietary zinc deficiency—resulted in reduced GI barrier function along with decreased occludin phosphorylation at sites known to be important for TJ assembly [254].

The GI barrier compromise associated with alcohol intake is well known to be exacerbated by zinc deficiency. Zinc deprivation itself associated with epithelial barrier disruption and added to the disruption induced by alcohol. Moreover, alcohol intake in mice not only compromised the ileal barrier but also lowered the tissue zinc concentration [255]. This pattern of alcohol intake lowering the tissue zinc levels—thus, the zinc deficiency adding to the barrier compromise induced by alcohol intake—was also found to exist outside the GI tract, manifesting itself in airway epithelia as well [256].

Zinc deficiency can result from low dietary zinc intake (e.g., certain vegan diets or malnutrition generally) to non-zinc dietary components (e.g., high phytate content in the diet), but recent research suggests that chronic proton pump inhibitor (PPI) use might also be causal. Zinc uptake has been shown to be inhibited by PPI use in humans [257,258] as well as rodents [259], although this phenomenon is not seen in all studies [260]. It also does not necessarily follow that interference with uptake translates to lower systemic zinc levels [261] although combination of PPI therapy with another disorder, such as chronic kidney disease, is more likely to present with a zinc deficiency systemically [262].

### 3.2. Vitamin A Deficiency

A recent review on Vitamins A and D in intestinal homeostasis concludes that these vitamins definitely play a regulatory role on TJ components [263]. Research on the GI epithelia of fish has shown that Vitamin A deficiency consistently induced downregulation of both an array of mRNA transcripts as well as actual TJ proteins [264,265]. In rats, Vitamin A deficiency reduced ZO-2 in colonic epithelial cell layers [266]. In corneal epithelium, loss of Notch1—a condition mimicking decreased Vitamin A downstream signaling—delayed ZO-1 incorporation into TJs and impaired barrier function [267]. Chung [268] showed that Vitamin A receptor deficiency delayed ZO-1 incorporation into Sertoli cell TJs. Huang [269] earlier found that Vitamin A deficiency led to compromised Sertoli cell TJ barrier function. Vitamin A deficiency in airway epithelial cell culture models shifted the actual differentiation of the cells from a mucosecretory histology to a stratified squamous histology, though the impact on barrier function was not addressed [270].

As was true with zinc deficiency, Vitamin A deficiency exacerbates other pathophysiological states that themselves compromise barrier function. Vitamin A deficiency in rats increased severity of lactose-induced diarrhea and the accompanying downregulation of intestinal TJ proteins [271]. A remarkable study of the fecal microbiota of children with Vitamin A deficiency has shown that their microbiome—transplanted to rodent colons—induced colonic barrier dysfunction in germ-free mice, including downregulation of the barrier proteins, occludin and claudin-1 [272].

However, the published literature is not in complete agreement. Ismail and Morales [273] disputed the findings of Huang [269], showing instead that Vitamin A deficiency in rats did not compromise Sertoli TJ barrier function. Gorodeski [274] actually claimed that a Vitamin A-deficient culture medium increased the barrier function of cervical epithelial cell layers.

### 3.3. Vitamin D Deficiency

Vitamin D deficiency can increase the risk of a variety of diseases that include an epithelial or endothelial barrier compromise as part of their etiology. As one example, Vitamin D deficiency has been linked to increased severity and acquisition of viral infections such as COVID-19 [275]. Lower serum Vitamin D levels have been reported in COVID-positive patients compared to COVID-negative patients [276]. Diseases not associated with viral infection but exacerbated by low Vitamin D levels include osteomalacia and rickets [277]. A detailed review by Ames [278] describes many other disease outcomes exacerbated by Vitamin D deficiency in African Americans. These include pregnancy complications, cancer, diabetes and asthma complications, some involving epithelial barrier compromise.

Many studies have associated a Vitamin D deficiency with specifically a leaky GI epithelial barrier. It is hypothesized that this leaky barrier can progress to different GI diseases such as IBD or celiac disease. In T_84_, a human intestinal epithelial cell line model, supplementation with calcitriol increased the expression of the Vitamin D receptor (VDR) and the formation of a complex with histone-deacetylase (HDAC11), which then promoted TJ formation. This type of interaction is decreased in Vitamin D receptor-deficient cells and leads to a leakier barrier [279]. Vitamin D-deficient mice showed an exaggerated increase in colon permeability in response to bacterial infection [280].

In intestinal epithelial tissues, Vitamin D deficiency has been associated with disorders that manifest impaired barrier function, such as UC and Crohn’s. In one study, lower serum Vitamin D levels were correlated with the severity of Crohn’s Disease (CD). Mucosal tissue from CD patients had lower expression of the TJ proteins occludin, claudin-1, ZO-1 and JAM-1 [281]. Kellermann et al. [282] speculated that low serum Vitamin D is linked to more severe cases of IBD due to factors causing leaky gut. Vitamin D deficiency in rodents has been shown to induce significant upregulation of the barrier-compromising protein, zonulin, elevation of serum proinflammatory cytokines, and a decrease in the intestinal barrier proteins, claudins -1, -3 and -7 [283]. Wei et al. [284] report that Vitamin D deficiency exacerbates TNBS-induced barrier dysfunction in mouse colon and attribute this exaggerated response to abnormal activation of the renin–angiotensin system, a pathway commonly activated in IBD.

The majority of studies have focused on the barrier effect of Vitamin D deficiency in specifically intestinal tissues, but other epithelial tissues can be affected as well. In the blood–brain barrier (BBB), a study completed with VDH^def^ mice showed that their BBB had reduced TJ expression after stroke, which could then potentially complicate recovery of the brain [285]. Vitamin D deficiency also plays a role in lung diseases such as asthma and COPD [286]. VDR-deficient mice were shown to have decreased mRNA and protein levels of claudin-2, claudin-4, and claudin-12. These changes caused by Vitamin D deficiency can be associated with lung permeability seen in pneumonia [287]. In at least one study of Vitamin D deficiency, supplementation was shown to reverse the negative effects of deficiency on the epithelial barrier regarding inflammation, an improvement accompanied by change in claudin-1 expression [288].

## 4. Can Elevated Micronutrient Levels (Supplementation) Improve Barrier Function?

### 4.1. Zinc Supplementation

Zinc has the largest associated published literature of any micronutrient on the topic of induced TJ changes and barrier enhancement. The majority of this literature focuses on the GI tract, not surprisingly since zinc has long been known to be an effective treatment for certain types of diarrhea [289,290,291]. Not surprisingly, many reviews have already been published on the topic. Hering and Schulzke [292] and Amasheh et al. [293] both describe zinc’s beneficial effects on GI barrier function in overall descriptions of micronutrient actions on barrier function. Zhou and Zhong [294] discuss the palliative effects of zinc on GI barrier function in the context of offsetting the barrier-compromising effects of alcoholic liver disease. Skrovanek et al. [295] also focus specifically on zinc and barrier function but in the more general context of miscellaneous GI diseases.

There are mainly four types of research studies that have been performed involving zinc-induced changes in GI barrier function and TJs. The first two categories are those focused on the zinc effects on controlling (basal) epithelial barrier function, performed using either epithelial cell culture models, such as Caco-2 or T_84_, or performed in animal models. In the third and fourth categories are those studies looking at the zinc effects on barrier compromise brought about by some pathophysiological condition or pathogenic agent, and again using either cell culture or animal models.

In the first category, zinc in the 50–100 µM range has been reliably observed to increase TER across normal Caco-2 cell layers. Wang et al. [4] and Valenzano et al. [296] did not, however, observe an accompanying change in occludin or claudin-1 expression, nor was there an accompanying decrease in transepithelial ^14^C-mannitol diffusion, although a change in the localization of claudins -2 and -7 was observed. Shao et al. [297] also observed a zinc-induced increase in TER across Caco-2 cell layers while also seeing increased ZO-1 expression, changes they observed were being transduced by the PI3K/AKT pathway. Furthermore, in Caco-2 cell layers, zinc was found to reduce the bulk transepithelial fluid flow, including lower transepithelial diffusion of FITC-dextran (4 kDa) [298]. A somewhat related study in Caco-2 showed that downregulating the ZnR/GPR39 zinc-sensing receptor decreased TER and reduced the expression of occludin and ZO-1 [299].

In the second category, numerous animal model studies of the zinc effects on basal GI barrier function have been done in piglets. While also increasing occludin and ZO-1 expression in ileal mucosa, zinc dietary supplementation decreased the L/M ratio in the urine, thereby signifying reduced small bowel leakiness [300]. Hu et al. [5] made a similar observation concerning a zinc-induced reduction in FITC-dextran (4 kDa) jejunal leak and upregulated occludin, ZO-1 and claudin-1 expression. Grilli et al. [301] likewise observed increased occludin and ZO-1 content. Peng et al. [302] and Zhu et al. [303] observed this as well as decreased spontaneous incidence of diarrhea. Wang et al. [304] noted that there were also accompanying zinc-induced changes in the GI microbiome. To illustrate how varied the models showing GI barrier improvement by zinc supplementation are, and how near universal the phenomenon is, zinc-induced upregulation of occludin, ZO-1 and claudin-1 has also been seen in the jejunum of ducks [305]. Likewise, zinc-induced upregulation of occludin and ZO-1 as well as reducing the L/M excretion ratio has been observed for rat small intestine [306]. In mouse small intestine, knockout of the ZIP14 zinc transporter was observed to increase the FITC-dextran leak and alter the phosphorylation state of occludin [254]. It is worth pointing out, however, that the zinc action on the GI tract, its fluid movements and diarrhea is not restricted just to the TJ, as zinc action on the ileal short circuit current through inhibition of basal-lateral potassium channels can also be in play [307].

In the third category, zinc has been shown to dramatically protect from the barrier compromise produced by microbes and microbial toxins. Ranaldi et al. [308] recorded that zinc supplementation of the Caco-2 cell layers prevented the barrier compromise caused by Ochratoxin A. Similarly, zinc oxide afforded protection from the Caco-2 barrier damage caused by enterotoxigenic *E. coli* [309]. This was later extended to protection from Cryptosporidium-induced barrier compromise accompanied by reduced claudin-4 expression, both being improved by zinc treatment [310]. Shigella-induced damage of T_84_ cell layers—reduced TER, increasing the FITC-dextran leak and mislocalization of claudins -2 and -4—was also reduced by zinc [311]. A similar observation was made for Salmonella-induced compromise of Caco-2 cell layers and downregulation of occludin and ZO-1, these also being partially redressed by zinc treatment [297].

The fourth type of research study, animal model studies where zinc reduces or blocks the barrier-compromising action of another agent, has a substantial published literature. A piglet GI study of chemically induced colitis showed that zinc could partially block the effects of the acetic acid-induced elevation of the FITC-dextran leak accompanied by decreased expression of occludin, claudin-1 and ZO-1 [312]. In dinitro-benzene-sulfonic acid-induced colitis in rats, individually leaky TJs could be visualized in electron microscopy, a phenomenon that was reduced by dietary zinc treatment [313]. Ethanol treatment increased the transepithelial permeability in rat ileum while also decreasing occludin, claudin-1 and ZO-1, and this action was also partially blocked by zinc [314]. Simple malnutrition—produced by a protein-deficient diet—led to barrier compromise and altered TJ structure in freeze fracture electron microscopy, and this too was counteracted partially by zinc treatment [315]. Another study in duck intestine reported zinc’s ability to partially block lipopolysaccharide (LPS) effects on barrier function and TJ proteins (occludin, claudin-1, ZO-1) [316]. Finally, zinc effects on specifically microbially-induced GI barrier leak in animal model studies is a similar story. Alpha-hemolysin-producing *E. coli* induced barrier compromise in pig colon along with claudin-4 and -5 downregulation, and this action was countered by zinc [152]. Similarly, *Clostridium perfringens*-induced jejunal barrier compromise and tissue inflammation in chickens was reduced by dietary zinc [317].

Before closing the discussion on zinc and GI barrier function, special mention of a study performed in humans (a class of studies dealt with in its own section below) merits attention here. The well-described GI barrier leak associated with heavy exercise in humans was observed to be associated with changes in the small intestinal occludin phosphorylation state. Both phenomena were found to be partially opposed by 14-day, dietary zinc treatment [318].

The physiologically beneficial action of zinc on barrier function is by no means restricted to the GI tract. Zinc was observed to increase the TER and decrease the paracellular leak in a human gingival epithelial cell culture model [3]. A combination of zinc and arginine was observed to protect against TNF-α-driven barrier compromise in another gingival epithelial barrier function study by similar methods [319]. The zinc transport protein, ZIP9, is known to mediate testosterone regulation of TJ formation in Sertoli cells [320]. Zinc has been reported to counter the blood–brain barrier leak (along with occludin downregulation) caused by aluminum exposure in rats [321]. Worth noting here is that zinc was reported to actually induce leak in rat blood–brain barrier models, but this was observed in the context of oxygen deprivation/ischemic conditions [322]. Zinc was without effect on basal barrier function in a human airway epithelial cell culture model (16HBE) (Callaghan [14]), however, it was showm in rat airway epithelial barriers made leaky by ethanol ingestion, that zinc supplementation partially countered this effect [256]. In a porcine renal cell culture model (LLC-PK_1_), zinc improved the basal barrier function (increased TER, decreased transepithelial ^14^C-mannitol diffusion), but had no effect on the expression levels of occludin or several claudins [323]. In the MDCK renal cell culture model, short-term zinc treatment also increased TER as well as dilution potentials and bi-ionic potentials, but likewise exerted no observed effect on occludin or ZO-1 [324]. In rat kidney, zinc was observed to protect against aberrant TJ changes induced by cadmium [325]. It is worth mentioning that the zinc effects seen in the LLC-PK_1_ renal cell culture model were potentiated by simultaneous treatment with the flavonoid quercetin [326]. The nutraceutical berberine—itself an effective modulator of GI epithelial TJs and enhancer of barrier function—may have its beneficial barrier effects mediated by increased intracellular zinc [327].

### 4.2. Vitamin A Supplementation

As was true for zinc, numerous reviews concerning Vitamin A and improved barrier function have been produced. Three focus specifically on GI barrier function. de Medeiros et al. [328] approach the topic from the point of view of an intestinal barrier compromised by malnutrition and enteric pathogens in a pediatric population. Abdelhamid and Luo [329] focus on autoimmune disease compromise of GI barrier function and the intertwined effects of Vitamin A and Vitamin A-modified GI microbiota in offsetting that compromise. The review by Cantorna et al. [263] examines GI barrier function modulation by Vitamins A and D in the context of the GI microbiota and the mucosal lymphoid cell population.

Specific research studies have used both cell culture and animal tissue models to show barrier improvement by Vitamin A by means of both upregulations of barrier proteins as well as improved measures of actual transepithelial barrier parameters. Again, as was true for zinc, the majority of these studies focus on the GI epithelial barrier. Increased TER and increased ZO-2 protein content were induced by trans-retinoic acid treatment of Caco-2 cell layers, along with decreased secretion of the barrier antagonist protein, zonulin [266]. In human intestinal organoids derived from human pluripotent stem cells, trans-retinoic acid increased the ZO-1 content and TER and also decreased the transepithelial leak of fluorescently labeled dextran [330]. A study in piglets by Wang et al. [331] did not address intestine barrier function per se but did observe that Vitamin A treatment increased parameters such as villous height, crypt depth and number of villi per unit area, findings that would suggest barrier improvement. In human colon adenocarcinoma cells in culture, trans-retinoic acid increased junctional subcellular localization of ZO-1 and occludin, an effect possibly mediated by downregulation of myosin light chain kinase [332].

Vitamin A was also effective in partially or fully restoring the GI barrier function that was compromised by different types of disease. Filteau et al. [333] reported that although Vitamin A was ineffective in improving the barrier function of healthy GI mucosa of infants, it significantly reduced the barrier compromise in an HIV-infected infant population. Lima et al. [334] observed a Vitamin A-induced decrease in lactulose and mannitol leakage in a pediatric population, a change that was associated moreover with reduced Giardia infection. In mice, the intestinal content of ZO-1, claudin-1 and occludin was reduced by endotoxin exposure, an effect that was partially offset by Vitamin A pretreatment. Aberrant subcellular localization of claudin-1 was also improved by Vitamin A, both effects possibly relating to a Vitamin A reduction in tissue levels of TNF-α and Interleukin-6 [335]. In Caco-2 cell layers challenged by endotoxin, pretreatment with Vitamin A significantly offset the ensuant TER reduction and reduced the decrease of occludin, claudin-1 and ZO-1 expression produced by endotoxin [336]. A similar finding was made in the HT-29 model with LPS and Vitamin A [337]. Similarly, Caco-2 cell layers made leaky (decreased TER) by exposure to Clostridium difficile toxin A were less leaky in the presence of Vitamin A [338].

There is substantial evidence of Vitamin A-induced barrier function enhancement in non-GI models as well. In the MDCK renal epithelial cell culture model, Vitamin A decreased the transepithelial inulin leak, while increasing expression of occludin, ZO-1 and claudins 1–4 [339]. Diabetes-induced reduction of occludin, claudin-2 and claudin-5 in rat kidney proximal tubules was partially blunted by Vitamin A treatment [340]. In the human airway epithelial cell culture model, 16HBE, Vitamin A improved basal barrier function (increased TER, reduced mannitol leak, increased levels of claudin-4), while also reducing the TNF-α-induced compromise of barrier function [14]. Lochbaum et al. [341], however, reported a negative effect of Vitamin A on barrier function of the human epithelial pulmonary adenocarcinoma cell line model, NCI-H441. In oral epithelial models, Hatakeyama [342] reported increased expression of occludin, ZO-1 and claudin-4 as a result of Vitamin A treatment. Groeger et al. [343] and Rybakovsky et al. [3] both reported Vitamin A-mediated improvement of gingival barrier function (increased TER, decreased mannitol leak), also with increased claudin-4 expression. Vitamin A has also been observed to modulate TJ proteins in the blood–testes barrier (occludin, ZO-1 and claudin-11 upregulation) and in brain capillary endothelia (occludin and claudin-5 upregulation) as well [344,345]. In lung endothelia, Vitamin A increased TER and reduced transendothelial albumin leak while also increasing ZO-1 and 7H6 junctional protein expression [346]. Retinal pigmented epithelial (RPE) cell layers showed improved barrier function (increased TER) after Vitamin A treatment while also increasing occludin and ZO-1 [347]. 

In effects that suggest a differentiation-inducing action of Vitamin A, Kubota et al. [348] reported upregulation of TJ proteins (occludin, claudin-6 and claudin-7), decreased transepithelial leak of 10 kDa dextran and increased complexity of TJ ultrastructure in F-9 embryonal carcinoma cell layers. Tobioka et al. and Retana et al. [349,350] reported a Vitamin A-induced increase in TJ protein expression (occludin) and improved barrier function (increased TER) in mesothelial cell layers. Vitamin A has also induced TJ formation in epidermal keratinocyte cultures while modulating TJ protein expression [351,352].

Vitamin A action, however, has been reported to compromise barrier function in certain models. In addition to the airway model reported above by Lochbaum et al. [341], this negative action has been reported for cervical epithelia by Gorodeski et al. [353] as well as the Caco-2 intestinal model under serum-free conditions [354].

### 4.3. Vitamin D Supplementation

In the previous section, we reported how a Vitamin D deficiency can induce epithelial barrier leak in a variety of tissues/cell types. It is not necessarily true, however, that Vitamin D *supplementation* would do the converse, namely, *improve* barrier function. However, in many cases, this in fact is true. It suggests that concentrations/intakes of Vitamin D above the RDA levels would be efficacious in reducing barrier leak in certain tissues.

Excellent reviews targeting this subject of Vitamin D supplementation and improved GI barrier function exist. Gubatan and Moss [355] approach the topic from the viewpoint of the inflamed barrier in ulcerative colitis (UC), describing how Vitamin D can protect the barrier by modifying TJ proteins and reducing rates of epithelial apoptosis. They also point out that UC patients frequently present with Vitamin D deficiency, as well as the fact that Vitamin D also modifies the GI microbiome. Cantorna et al. [263] in addition describe studies with the Vitamin D receptor as well as Vitamin D but reaching the same conclusion. Noriega and Savelkoul [356] describe how the Vitamin D status during gestation and early life may be mitigating allergy susceptibility possibly through airway and GI epithelial barrier functionality. Battistini et al. [357] review publications showing that Vitamin D deficiency and supplementation may achieve their effects on GI barrier function in part through Vitamin D effects on the GI microbiome.

As indicated in the above reviews, most of the published literature on this phenomenon of Vitamin D improvement/protection of barrier function comes again from studies on the GI tract. The majority also focuses on a prominent disease associated with barrier leak, namely, Inflammatory Bowel Disease (IBD), and particularly UC. Epithelial cell culture models of UC typically involve proinflammatory cytokine treatment of Caco-2 or other GI epithelial cell culture models. Du et al. [358] showed that Vitamin D can inhibit TNF-α-induced increases in Caco-2 cell layer permeability as well as MLCK activation. Kong et al. [359] earlier showed that Vitamin D could enhance basal Caco-2 barrier function as seen by increased TER and modifications of TJ proteins. Vitamin D Receptor (VDR) knockdown had the opposite effect. Lee et al. [360] noted that LPS treatment of IEC-18 cell layers induced TNF-α synthesis and barrier leak accompanied by decreased ZO-1 and claudin-2, all of which were reversed by Vitamin D. In animal studies, Kong et al. [359] observed that knockout of VDR increased the severity of DSS-induced colitis. A similar finding was reported by (Zhao, et al. [361]). Liu et al. [362] observed that Vitamin D reduced the severity of 2,4,6-trinitrobenzene sulfonic acid (TNBS)-induced colitis in mice, increasing the barrier properties and the occludin and ZO-1 levels. Du et al. [358] reported a similar finding along with Vitamin D reducing MLCK activation. Chatterjee et al. [363] used Vitamin D treatment or VDR overexpression to decrease susceptibility to chemically induced colitis, which correlated with overexpression of claudin-15, whose promoter is targeted by Vitamin D. The claudin-2 promoter is also held to be directly regulated by Vitamin D [364]. LPS-induced GI inflammation was also reduced by Vitamin D, which restored the reduced levels of claudins -1 and -5 [365]. A combined study of Vitamin D and Vitamin C supplementation has suggested that effects on claudin-2 levels and barrier improvement may be mediated through Notch signaling [366].

There are also non-UC studies involving the GI tract and Vitamin D. Caco-2 cell layers incubated with enterohemorrhagic *E. coli* manifested impaired barrier function, a condition reversed by simultaneous incubation with Vitamin D [367]. *Campylobacter jejuni* impairment of intestinal barrier function (TER and fluorescein permeability) in mice was partially reversed by Vitamin D supplementation [147]. Caco-2 cell layers made leaky by ethanol exposure were partially protected by preincubation with Vitamin D, a phenomenon also observed in mouse intestine [368]. In a celiac disease-related model, Caco-2 cell layers exposed to gliadin peptides exhibited increased transepithelial leak to FITC dextran 4000, which was partially blocked by simultaneous incubation with Vitamin D [369]. CCl_4_-induced cirrhosis in mice is accompanied by intestinal barrier compromise with increased bacterial translocation, but simultaneous treatment with Vitamin D was observed to preserve intestinal barrier function [370]. A similar Vitamin D protection of intestinal barrier function was observed regarding the increased intestinal bacterial translocation induced by severe burn injury in mice [371]. Decreased rat GI barrier function and increased bacterial translocation in cirrhotic rats was partially reversed by Vitamin D supplementation, along with upregulation of occludin and claudin-1 [372].

This overall theme of Vitamin D protection of epithelial barrier function also holds true in various epithelial tissues other than the GI tract. In a human retinal pigment epithelium cell culture model, LPS and TNF-α-induced barrier compromise and TJ alteration were offset by Vitamin D exposure [373]. Dust mite-induced rhinitis induces barrier leak in human nasal epithelial cell layers, a condition partially countered by Vitamin D [246]. Mouse urinary bladder epithelial barriers compromised by *E. coli* infection were partially protected by Vitamin D administration, reversing the infection-induced reduction in occludin and claudin-14 [374]. Partial VDR knockout in mice resulted in a more severely, LPS-compromised pulmonary epithelial barrier, with decreased occludin and ZO-1 expression, and this situation was alleviated by Vitamin D [375]. Mice exposed to the asthma-inducing agent, toluene diisocyanate, developed increased leak across their alveolar barriers along with decreased occludin expression, with both phenomena partially reversed by Vitamin D treatment or an ERK1/2 inhibitor [376]. Hypoxia-induced compromise of mouse brain capillary endothelia barriers (decreased TER, increased FITC-dextran [40 kDa] leak) along with decreased ZO-1, claudin-5 and occludin, was prevented in its entirety by Vitamin D treatment [377]. Finally, regarding the epidermal barrier, oral Vitamin D improvement of atopic dermatitis in humans is postulated to involve Vitamin D-improved barrier function [378].

In closing, it is worth pointing out that this beneficial effect of Vitamin D on barrier function is not exclusively about *decreased* permeability. This fact speaks to the degree of fine tuning that exists regarding the regulation of our epithelial barriers by Vitamin D and certain micronutrients in general. There exist certain electrolytes and nonelectrolytes whose physiological absorption/reabsorption in the GI tract and the kidney occur via the paracellular pathway, i.e., through the TJ barrier. Ca^++^ and Mg^++^ are prominent in this regard. It is very worth noting that Vitamin D treatment *increases* duodenal Ca^++^ absorption, in large part through the paracellular pathway, in a process believed to rely in part on upregulation of claudins -2 and -12 [379,380]. Vitamin D-induced increase in paracellular Ca^++^ diffusion has been verified in the Caco-2 cell culture model [381]. However, a warning on the Mg^++^ side is that Vitamin D transcriptionally repressed claudin-16 expression, and this has been observed to lead to increased Mg^++^ (and Ca^++^) excretion in urine [382].

## 5. Clinical Evidence for Elevated Micronutrient Levels as Therapeutic Strategies: Patient-Based Studies

The model for which there is the least amount of reported evidence for improved barrier function by micronutrients is the all-important patient-based clinical studies. These are also the studies (models) most fraught with issues of inherent variability emanating from individual differences in genetics, age, diet and medications—perhaps not a coincidence, then, that they are in scarcity. Camilleri [383] very validly points out in a recent review that it has never been conclusively proven that although micronutrient fortification of (intestinal) epithelial barrier leakiness is apparently quite real, conclusive evidence showing true amelioration of disease-related clinical manifestations by barrier-enhancing micronutrients has been elusive.

Though few studies appear to have been done, and inconclusiveness is an obvious conclusion throughout, evidence in this key model does nonetheless exist and is growing. DiGuilio et al. [384] have very recently reported that oral zinc administration does induce TJ protein changes (increased levels of claudins -3 and -5) along with general transcriptome changes suggestive of modified barrier function in healthy human duodenal mucosa (abstract presentation at annual meeting of the American Gastroenterological Association, 2021). However, functional barrier studies had not yet been performed. Other groups have, however, reported functional improvement of the GI barrier in humans by zinc. Sturniolo et al. [385] reported a reduction in the L/M permeability ratio by 40% in Crohn’s Disease patients treated with an oral zinc supplement for 8 weeks. Roy et al. and Alam et al. [386,387] had reported that zinc reduced the L/M ratios in children with acute or persistent microbial-driven diarrhea. Ryan et al. [388] had a similar finding with children manifesting diffuse enteropathy characterized by T-cell infiltration of small bowel mucosa. Using the very similar lactulose/rhamnose (L/R) permeability test, zinc-improved GI barrier function was also demonstrated in a pediatric population with gastroenteropathy [389]. In a healthy adult study, where vigorous exercise was used to elevate the L/R ratio, zinc carnosine was able to reduce the L/R ratio to near normal values [318]. An ex vivo study in asthmatic patients with *Dermatophagoides pteronyssinus* allergies showed that zinc supplementation reduced secreted levels of Interleukins -4 and -17, suggesting reduced systemic inflammation may partially account for zinc’s positive effects on barrier function [390].

Colon biopsy tissue from actively inflamed mucosa of UC patients showed a reduction of claudins -4 and -7 expression. After incubation (ex vivo) with Vitamin D, these expression levels were increased [391]. Again, in ex vivo studies with colon biopsies from UC patients, Vitamin D decreased the claudin-2 levels while increasing the claudin-4 levels [392]. In a study of Crohn’s Disease patients in China, serum levels of Vitamin D were not only lower than in healthy controls, but those levels inversely correlated with the Crohn’s Disease Activity Index in the patients, and directly correlated with levels of expression of TJ proteins (occludin, claudin-1, ZO-1 and JAM-A) in the GI mucosa [393]. Meckel et al. [394] also observed that Vitamin D levels in serum were inversely correlated with UC colon mucosal inflammation, and positively correlated with the colon biopsy occludin and ZO-1 levels. In a Vitamin D patient-based study not focused on GI barrier function, Dancer et al. [395] reported that lung water accumulation in Adult Respiratory Distress Syndrome (ARDS) was exacerbated in patients with Vitamin D deficiency and, in turn, reduced significantly by Vitamin D supplementation.

Filteau et al. [333] reported in infant small intestine that whereas Vitamin A treatment had no effect on basal barrier function, it did reduce HIV-mediated barrier compromise. Lima et al. [334] observed that Vitamin A treatment reduced the GI leakage of lactulose and mannitol individually (but no significant change in the L/M ratio) in a pediatric population (while also reducing incidence of Giardia infection). Thurnham et al. [396] reported that the Vitamin A treatment of infants reduced the increase in L/M that is associated with weaning.

Although not a micronutrient, the GI zonulin antagonist (drug), larazotide, has been—and is being—tested in humans, but has had mixed results regarding barrier function improvement across a variety of different patient populations [143].

## 6. Current Nutrition Guidelines and Consideration of Micronutrient Elevation as an Adjuvant Therapeutic

The concept or guideline of the Recommended Dietary Allowance (RDA) establishes the frame of reference by which health care workers judge the nutrient needs of a patient. The Institute of Medicine’s Food and Nutrition Board first introduced the RDA in the 1940s. Initially, reviewed every 5–10 years, the purpose of these specific nutrient standards was to prevent nutrient deficiencies and reduce nutrition-related chronic diseases. In 1997, the Dietary Reference Intakes (DRIs) were established, expanding upon the RDA index. The DRI is a more comprehensive term that includes the Estimated Average Requirement (EAR), the RDA, Adequate Intake (AI) and Tolerable Upper Intake Level (UL). The EAR is the amount of nutrient intake that meets the estimated nutrient needs of half of the individuals in a population group. The EAR is derived based on best available evidence from scientific studies and is used to develop the RDA. The DRIs continue to be reviewed and updated on a regular basis as sound evidence-based research becomes available. The current RDAs for zinc and Vitamin A were established in 2001, and for Vitamin D in 2011. The current RDA for zinc is 8 mg for females and 11 mg for males; for Vitamin A, 700 mcg (2333 IU) for females, 900 mcg (3000 IU) for males; and for Vitamin D, 15 mcg (600 IU) for adults up to 70 years old [397,398,399]. (See Table 1).

According to the Institute of Medicine, US Panel on Micronutrients, consumption of the quantity of the RDA of a specific nutrient should decrease one’s chance of developing a condition that is both associated with that nutrient and that negatively affects one’s functional status. The DRIs are conservatively established and cautiously revised as their sole purpose is to prevent deficiency and avoid toxicity on the population level. A gap exists in the published literature as to what nutrient level may provide a pharmacologic and therapeutic effect on specific disease states. In addition, it is important to recognize that the DRIs were established for healthy individuals consuming a normal oral diet and were never designed to be applied to those on nutrition support with or without an acute illness or inflammatory process [397,398,400].

**Table 1:** Current recommended adult daily oral and parenteral micronutrient requirements and content provided in multi-vitamin/trace products currently available in the US [398,401,402].

	Recommended Oral Requirements	Recommended Parenteral Requirements	Content Provided in Multi-Vitamin/Trace Products Currently Available in the US
Vitamin A	Male: 900 mcg or 3000 IU Female: 700 mcg or 2333 IU	990 mcg or 3300 IU	3300 IU per 10 mL in MVI
Vitamin D	Age 19–70 years: 15 mcg or 600 IU	5 mcg or 200 IU	200 IU per 10 mL in MVI
Zinc	Male: 11 mg Female: 8 mg	3–5 mg	varies between 3–5 mg based on product

MVI: injectable multivitamin.

There is much debate about how the RDA levels were established and how they can be revised. Only after much advocacy in 2011, the RDAs for both calcium and Vitamin D were increased in part to address the issue of bone fractures. Currently, the DRIs also include a Tolerable Upper Intake Level (UL). Researchers have advocated for the UL framework to be revised. Rather than a single point value, a description of the range of adverse effects merits being advocated for. Altering the hard stop UL may make higher doses of specific nutrients available and recommended for specific conditions as long as potential adverse effects are made known (as with any other medication) [398,400].

On a larger scale, the RDA system is complicated by a number of variables. First, there is no analytical model or established framework for assessing disease outcomes for most nutrients. Second, a regular review process for existing DRIs does not exist. Related to this, there is a lack of consistent financial funding from the government for DRI-related research and activities [398,400].

The current recommended parenteral doses of fat-soluble vitamins (e.g., Vitamins A and D) are approximately equal to the current oral RDAs for these vitamins. These parenteral recommendations are based on the assumption that in parenteral feeding, bioavailability will be greater, but requirements may be higher due to disease conditions affecting metabolic needs, malabsorption, and baseline deficiencies. Since the Institute of Medicine increased the oral RDA for Vitamin D back in 2011, there is concern as to how appropriate the current recommended parenteral Vitamin D dose may be [398,401,402].

In 2012, the American Society for Parenteral and Enteral Nutrition (ASPEN) published a position paper about the recommendations for changes in commercially available parenteral multivitamin and multi-trace element products. Following this, ASPEN published another paper in 2015 calling on nutrient manufacturers to produce products that adhere to their recommendations for these nutrients. Highlighting the growing awareness of a need to increase the daily allowances of certain micronutrients in at least certain pathophysiological states, the authors in two publications have, for example, urged the parenteral nutrition industry leaders and the Food and Drug Administration (FDA) to create a separate parenteral Vitamin D product. There are no current Vitamin D options/products available in the US for patients who are dependent upon parenteral nutrition and unable to tolerate oral vitamins. Vitamin D, like other micronutrients, is absent from parenteral nutrient admixtures, and is provided only at low levels in commercial multi-vitamin/trace product supplement mixtures. Providing adequate Vitamin D to patients requiring parenteral nutrition continues to create significant challenges for health professionals.

Individual parenteral zinc (on top of what is provided in m ulti-vitamin/trace product supplements) is available to be added to parenteral nutrition admixture when needs are greater than what a multi-trace element product includes. Similarly, individual intra-muscular Vitamin A products are available to be given to patients who have greater needs or develop deficiency. However, the general issue of elevating Vitamins A and D, as well as zinc, above the RDA levels in at least certain pathophysiological conditions remains open [397,398,401]. With regard to *enterally* fed individuals, most enteral formulations meet the DRIs of vitamins and minerals when given as a complete source of nutrition (i.e., provided in volumes of 1000–1500 mL/d or at least 1500 calories/day). However, note that in both cases—parenteral and enteral—the formulations are designed to achieve the DRI levels of daily micronutrient intakes.

**Figure 2:**
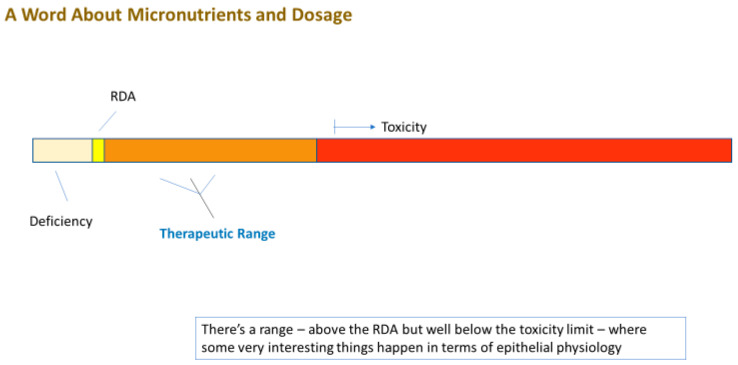
For many micronutrients, a therapeutic range can exist between the Recommended Daily Allowance (RDA) and concentrations/intakes that are toxic to the organism. In this range, activation of normally quiescent signaling pathways can occur, leading to beneficial physiological changes, among which is improved barrier function.

**Figure 3:**
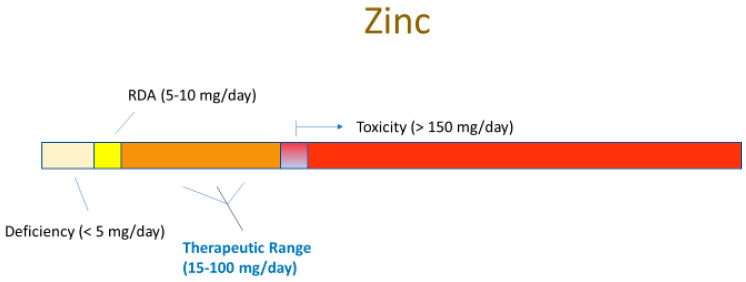
Various cell culture, animal and human studies indicate that intake of 20–100 mg of zinc per adult per day (which will result in transient blood levels in the 25–100 µM range) can achieve beneficial, zinc-induced physiological changes, among which may be TJ remodeling and barrier improvement.

The above discussion has dealt partly with the issue of the proper levels of Vitamin A, Vitamin D and zinc in non-healthy, tube-fed individuals. In other words, the discussion centers on the question of the *therapeutic* use of elevated micronutrient levels. Our review would question whether the currently delivered therapeutic levels (enteral and parenteral levels) are sufficiently high given the effectiveness of these three micronutrients in reducing disease-associated epithelial and endothelial barrier leaks (Figure 2 and Figure 3). There is, however, also the issue of a proper *prophylactic*; the health-supporting level of these micronutrients in healthy individuals and whether the current RDA levels adequately address those needs; and given the novelty, the evolving information about these micronutrients and epithelial/endothelial barrier leak, and how that may influence disease onset. This is especially true regarding infectious diseases whose pathogens must typically cross a barrier to fully infect an organism.

## 7. Summary

The conclusions from this review are fourfold: (1) epithelial and endothelial barrier compromise are endemic across a wide range of diseases; (2) deficiencies in specific micronutrients, such as zinc, Vitamin A and Vitamin D, can result in barrier leak and exacerbate both the leak caused by certain disease states and overall morbidity; (3) supplementation of these micronutrients can improve barrier function and reduce the impact of some disease states on barrier compromise; and (4) current nutritional guidelines do not acknowledge the ability of certain micronutrients to improve barrier function or reduce barrier compromise at levels above their current RDAs but below their toxicity limits. This is true for the general population but perhaps even more pointedly true for inpatients on supplemental feeding. These conclusions would seem to suggest that in certain medical conditions and disease states, supplementation of these micronutrients above the RDA levels may improve morbidity and have a genuine place in standard medical care. For the mindset of, “How significant can a micronutrient be?”, consider that even modest improvements in morbidity may be sufficient to, e.g., allow an individual’s systemic physiology to be sustained to the point where their immune response can suppress a specific microbial infection. We are moreover discussing substances whose toxicity profiles are very well known, and that tend to be not only relatively inexpensive to produce but are moderate to very stable in the field. All these considerations would seem to argue for the medical use of elevated micronutrients in various prophylactic as well as therapeutic/critical care settings as adjuvant approaches. In an emerging pandemic situation, such as COVID-19, the use of selected micronutrients to reduce barrier leak and stabilize physiology could be an early, rapid-use therapeutic utility as more substantive therapeutics are being developed. These micronutrients could also be useful prophylactically in strengthening epithelial defenses and reducing disease incidence.

## References

[1] Briefel R.R., Bialostosky K., Kennedy-Stephenson J., McDowell M.A., Ervin R.B., Wright J.D. (2000). Zinc Intake of the U.S. Population: Findings from the Third National Health and Nutrition Examination Survey, 1988–1994. J. Nutr..

[2] Gibson R.S., Hess S.Y., Hotz C., Brown K.H. (2008). Indicators of zinc status at the population level: A review of the evidence. Br. J. Nutr..

[3] Rybakovsky E., Valenzano M.C., Deis R., DiGuilio K.M., Thomas S., Mullin J.M. (2017). Improvement of Human-Oral-Epithelial-Barrier Function and of Tight Junctions by Micronutrients. J. Agric. Food Chem..

[4] Wang X., Valenzano M.C., Mercado J.M., Zurbach E.P., Mullin J.M. (2012). Zinc Supplementation Modifies Tight Junctions and Alters Barrier Function of CACO-2 Human Intestinal Epithelial Layers. Dig. Dis. Sci..

[5] Hu C., Song J., Li Y., Luan Z., Zhu K. (2013). Diosmectite–zinc oxide composite improves intestinal barrier function, modulates expression of pro-inflammatory cytokines and tight junction protein in early weaned pigs. Br. J. Nutr..

[6] Heinemann U., Schuetz A. (2019). Structural Features of Tight-Junction Proteins. Int. J. Mol. Sci..

[7] Suzuki T. (2020). Regulation of the intestinal barrier by nutrients: The role of tight junctions. Anim. Sci. J..

[8] Prot-Bertoye C., Houillier P. (2020). Claudins in Renal Physiology and Pathology. Genes.

[9] Slifer Z.M., Blikslager A.T. (2020). The Integral Role of Tight Junction Proteins in the Repair of Injured Intestinal Epithelium. Int. J. Mol. Sci..

[10] Dong D., Xie W., Liu M. (2020). Alteration of cell junctions during viral infection. Thorac. Cancer.

[11] Cong X., Kong W. (2020). Endothelial tight junctions and their regulatory signaling pathways in vascular homeostasis and disease. Cell. Signal..

[12] Hollander D., Kaunitz J.D. (2020). The “Leaky Gut”: Tight Junctions but Loose Associations?. Dig. Dis. Sci..

[13] Wessels I., Rolles B., Slusarenko A.J., Rink L. (2021). Zinc deficiency as a possible risk factor for increased susceptibility and severe progression of Corona Virus Disease 19. Br. J. Nutr..

[14] Callaghan P.J., Ferrick B., Rybakovsky E., Thomas S., Mullin J.M. (2020). Epithelial barrier function properties of the 16HBE14o- human bronchial epithelial cell culture model. Biosci. Rep..

[15] Kuo W.-T., Shen L., Zuo L., Shashikanth N., Ong M.D.M., Wu L., Zha J., Edelblum K.L., Wang Y., Wang Y. (2019). Inflammation-induced Occludin Downregulation Limits Epithelial Apoptosis by Suppressing Caspase-3 Expression. Gastroenterology.

[16] Rybakovsky E., Buleza N.B., Hoxha K., DiGuilio K.M., McCluskey E.S., Friday C.L., Callaghan P.J., Moskalenko D.V., Zuo B., Thomas S. (2018). Spontaneous and cytokine-induced hole formation in epithelial cell layers: Implications for barrier function studies with the gingival cell culture, Gie-3B11, and other epithelial models. Trends Cell Mol. Biol..

[17] Al-Sadi R., Guo S., Ye D., Rawat M., Ma T.Y. (2016). TNF-α Modulation of Intestinal Tight Junction Permeability Is Mediated by NIK/IKK-α Axis Activation of the Canonical NF-κB Pathway. Am. J. Pathol..

[18] Al-Sadi R., Guo S., Ye D., Ma T.Y. (2013). TNF-α Modulation of Intestinal Epithelial Tight Junction Barrier Is Regulated by ERK1/2 Activation of Elk-1. Am. J. Pathol..

[19] Petecchia L., Sabatini F., Usai C., Caci E., Varesio L., Rossi G.A. (2012). Cytokines induce tight junction disassembly in airway cells via an EGFR-dependent MAPK/ERK1/2-pathway. Lab. Investig..

[20] Mullin J., Snock K.V. (1990). Effect of tumor necrosis factor on epithelial tight junctions and transepithelial permeability. Cancer Res..

[21] Fink M.P. (2003). Intestinal epithelial hyperpermeability: Update on the pathogenesis of gut mucosal barrier dysfunction in critical illness. Curr. Opin. Crit. Care.

[22] Fink M.P., Delude R.L. (2005). Epithelial Barrier Dysfunction: A Unifying Theme to Explain the Pathogenesis of Multiple Organ Dysfunction at the Cellular Level. Crit. Care Clin..

[23] Huang Y., Mao Q., Shi X., Cong X., Zhang Y., Wu L., Yu G., Xiang R. (2020). Disruption of tight junctions contributes to hyposalivation of salivary glands in a mouse model of type 2 diabetes. J. Anat..

[24] Gu M., Mei X.-L., Zhao Y.-N. (2021). Sepsis and Cerebral Dysfunction: BBB Damage, Neuroinflammation, Oxidative Stress, Apoptosis and Autophagy as Key Mediators and the Potential Therapeutic Approaches. Neurotox. Res..

[25] Gonzales J., Lucas R., Verin A. (2015). The Acute Respiratory Distress Syndrome: Mechanisms and Perspective Therapeutic Approaches. Austin J. Vasc. Med..

[26] Rodrigues S.F., Granger D.N. (2015). Blood cells and endothelial barrier function. Tissue Barriers.

[27] Mariano F., Cantaluppi V., Stella M., Romanazzi G.M., Assenzio B., Cairo M., Biancone L., Triolo G., Ranieri V.M., Camussi G. (2008). Circulating plasma factors induce tubular and glomerular alterations in septic burns patients. Crit. Care.

[28] Zhao G.-J., Li D., Zhao Q., Lian J., Hu T.-T., Hong G.-L., Yao Y.-M., Lu Z. (2016). Prognostic Value of Plasma Tight-Junction Proteins for Sepsis in Emergency Department: An Observational Study. Shock.

[29] Assimakopoulos S.F., Akinosoglou K., de Lastic A.-L., Skintzi A., Mouzaki A., Gogos C.A. (2020). The Prognostic Value of Endotoxemia and Intestinal Barrier Biomarker ZO-1 in Bacteremic Sepsis. Am. J. Med. Sci..

[30] Mankertz J., Schulzke J.-D. (2007). Altered permeability in inflammatory bowel disease: Pathophysiology and clinical implications. Curr. Opin. Gastroenterol..

[31] Fakhoury M., Negrulj R., Mooranian A., Al-Salami H. (2014). Inflammatory bowel disease: Clinical aspects and treatments. J. Inflamm. Res..

[32] Hollander D. (1988). Crohn’s disease--a permeability disorder of the tight junction?. Gut.

[33] Schulzke J.D., Ploeger S., Amasheh M., Fromm A., Zeissig S., Troeger H., Richter J., Bojarski C., Schumann M., Fromm M. (2009). Epithelial tight junctions in intestinal inflammation. Ann. N. Y. Acad. Sci..

[34] Luettig J., Rosenthal R., Barmeyer C., Schulzke J.D. (2015). Claudin-2 as a mediator of leaky gut barrier during intestinal inflammation. Tissue Barriers.

[35] Teshima C., Dieleman L.A., Meddings J.B. (2012). Abnormal intestinal permeability in Crohn’s disease pathogenesis. Ann. N. Y. Acad. Sci..

[36] Takeuchi K., Maiden L., Bjarnason I. (2004). Genetic aspects of intestinal permeability in inflammatory bowel disease. Novartis Found Symp..

[37] Luissint A.-C., Parkos C.A., Nusrat A. (2016). Inflammation and the Intestinal Barrier: Leukocyte–Epithelial Cell Interactions, Cell Junction Remodeling, and Mucosal Repair. Gastroenterology.

[38] Bruewer M., Samarin S., Nusrat A. (2006). Inflammatory Bowel Disease and the Apical Junctional Complex. Ann. N. Y. Acad. Sci..

[39] Shen L., Turner J.R. (2006). Role of Epithelial Cells in Initiation and Propagation of Intestinal Inflammation. Eliminating the static: Tight junction dynamics exposed. Am. J. Physiol. Gastrointest. Liver Physiol..

[40] Odenwald M.A., Turner J.R. (2013). Intestinal Permeability Defects: Is It Time to Treat?. Clin. Gastroenterol. Hepatol..

[41] Zhu L., Han J., Li L., Wang Y., Li Y., Zhang S. (2019). Claudin Family Participates in the Pathogenesis of Inflammatory Bowel Diseases and Colitis-Associated Colorectal Cancer. Front. Immunol..

[42] Landy J., Ronde E., English N., Clark S.K., Hart A.L., Knight S.C., Ciclitira P.J., Al-Hassi H.O. (2016). Tight junctions in inflammatory bowel diseases and inflammatory bowel disease associated colorectal cancer. World J. Gastroenterol..

[43] Fries W., Belvedere A., Vetrano S. (2013). Sealing the broken barrier in IBD: Intestinal permeability, epithelial cells and junctions. Curr. Drug Targets.

[44] Larabi A., Barnich N., Nguyen H.T.T. (2020). New insights into the interplay between autophagy, gut microbiota and inflammatory responses in IBD. Autophagy.

[45] Marin M.L., Greenstein A.J., Geller S.A., Gordon R.E., Aufses A.H. (1983). A freeze fracture study of Crohn’s disease of the terminal ileum: Changes in epithelial tight junction organization. Am. J. Gastroenterol..

[46] Schmitz H., Barmeyer C., Fromm M., Runkel N., Foss H.-D., Bentzel C.J., Riecken E.-O., Schulzke J.-D. (1999). Altered tight junction structure contributes to the impaired epithelial barrier function in ulcerative colitis. Gastroenterology.

[47] Gitter A.H., Wullstein F., Fromm M., Schulzke J.D. (2001). Epithelial barrier defects in ulcerative colitis: Characterization and quantification by electrophysiological imaging. Gastroenterology.

[48] Oshima T., Miwa H., Joh T. (2008). Changes in the expression of claudins in active ulcerative colitis. J. Gastroenterol. Hepatol..

[49] Zeissig S., Bürgel N., Günzel D., Richter J., Mankertz J., Wahnschaffe U., Kroesen A.J., Zeitz M., Fromm M., Schulzke J.D. (2007). Changes in expression and distribution of claudin 2, 5 and 8 lead to discontinuous tight junctions and barrier dysfunction in active Crohn’s disease. Gut.

[50] Das P., Goswami P., Das T.K., Nag T., Sreenivas V., Ahuja V., Panda S.K., Gupta S.D., Makharia G.K. (2012). Comparative tight junction protein expressions in colonic Crohn’s disease, ulcerative colitis, and tuberculosis: A new perspective. Virchows Arch..

[51] Lameris A.L., Huybers S., Kaukinen K., Mäkelä T.H., Bindels R.J., Hoenderop J.G., Nevalainen P.I. (2013). Expression profiling of claudins in the human gastrointestinal tract in health and during inflammatory bowel disease. Scand. J. Gastroenterol..

[52] Xu C.-M., Li X.-M., Qin B.-Z., Liu B. (2016). Effect of tight junction protein of intestinal epithelium and permeability of colonic mucosa in pathogenesis of injured colonic barrier during chronic recovery stage of rats with inflammatory bowel disease. Asian Pac. J. Trop. Med..

[53] Zwiers A., Fuss I.J., Leijen S., Mulder C.J., Kraal G., Bouma G. (2008). Increased expression of the tight junction molecule claudin-18 A1 in both experimental colitis and ulcerative colitis. Inflamm. Bowel Dis..

[54] Erakovic Haber V., Cuzic S., Dominis Kramaric M., Hrvacic B., Polancec D., Banic M., Glojnaric I. (2015). Claudin expression in animal models of IBD and human disease. New Horiz. Transl. Med..

[55] Caviglia G.P., Dughera F., Ribaldone D.G., Rosso C., Abate M.L., Pellicano R., Bresso F., Smedile A., Saracco G.M., Astegiano M. (2019). Serum zonulin in patients with inflammatory bowel disease: A pilot study. Minerva Med..

[56] Arrieta M.C., Madsen K., Doyle J., Meddings J. (2008). Reducing small intestinal permeability attenuates colitis in the IL10 gene-deficient mouse. Gut.

[57] Olson T.S., Reuter B.K., Scott K.G.-E., Morris M.A., Wang X.-M., Hancock L.N., Burcin T.L., Cohn S.M., Ernst P., Cominelli F. (2006). The primary defect in experimental ileitis originates from a nonhematopoietic source. J. Exp. Med..

[58] Poritz L.S., Garver K.I., Green C., Fitzpatrick L., Ruggiero F., Koltun W.A. (2007). Loss of the Tight Junction Protein ZO-1 in Dextran Sulfate Sodium Induced Colitis. J. Surg. Res..

[59] Poritz L.S., Harris L.R., Kelly A.A., Koltun W.A. (2011). Increase in the Tight Junction Protein Claudin-1 in Intestinal Inflammation. Dig. Dis. Sci..

[60] Kucharzik T., Walsh S.V., Chen J., Parkos C.A., Nusrat A. (2001). Neutrophil Transmigration in Inflammatory Bowel Disease Is Associated with Differential Expression of Epithelial Intercellular Junction Proteins. Am. J. Pathol..

[61] Soderholm J.D., Olaison G., Peterson K.H., Franzén L., Lindmark T., Wirén M., Tagesson C., Sjödahl R. (2002). Augmented increase in tight junction permeability by luminal stimuli in the non-inflamed ileum of Crohn’s disease. Gut.

[62] Wyatt J., Vogelsang H., Hübl W., Waldhoer T., Lochs H. (1993). Intestinal permeability and the prediction of relapse in Crohri’s disease. Lancet.

[63] D’Incà R., Di Leo V., Corrao G., Martines D., D’Odorico A., Mestriner C., Venturi C., Longo G., Sturniolo G.C. (1999). Intestinal permeability test as a predictor of clinical course in Crohn’s disease. Am. J. Gastroenterol..

[64] Arnott I.D.R., Kingstone K., Ghosh S. (2000). Abnormal Intestinal Permeability Predicts Relapse in Inactive Crohn Disease. Scand. J. Gastroenterol..

[65] Chang J., Leong R.W., Wasinger V.C., Ip M., Yang M., Phan T.G. (2017). Impaired Intestinal Permeability Contributes to Ongoing Bowel Symptoms in Patients with Inflammatory Bowel Disease and Mucosal Healing. Gastroenterology.

[66] Hollander D., Vadheim C.M., Brettholz E., Petersen G.M., Delahunty T., Rotter J.I. (1986). Increased Intestinal Permeability in Patients with Crohn’s Disease and Their Relatives. A possible etiologic factor. Ann. Intern. Med..

[67] Teshima C.W., Goodman K.J., El-Kalla M., Turk S., El-Matary W., Valcheva R., Danchak R., Gordon M., Ho P., Mullins A. (2017). Increased Intestinal Permeability in Relatives of Patients with Crohn’s Disease Is Not Associated with Small Bowel Ulcerations. Clin. Gastroenterol. Hepatol..

[68] May G.R., Sutherland L.R., Meddings J.B. (1993). Is small intestinal permeability really increased in relatives of patients with Crohn’s disease?. Gastroenterology.

[69] Peeters M., Geypens B., Claus D., Nevens H., Ghoos Y., Verbeke G., Baert F., Vermeire S., Vlietinck R., Rutgeerts P. (1997). Clustering of increased small intestinal permeability in families with Crohn’s disease. Gastroenterology.

[70] Turpin W., Lee S.H., Raygoza Garay J.A., Madsen K.L., Meddings J.B., Bedrani L., Power N., Espin-Garcia O., Xu W., Smith M.I. (2020). Increased Intestinal Permeability Is Associated with Later Development of Crohn’s Disease. Gastroenterology.

[71] Irvine E., Marshall J. (2000). Increased intestinal permeability precedes the onset of Crohn’s disease in a subject with familial risk. Gastroenterology.

[72] Anderson C.A., Boucher G., Lees C.W., Franke A., D’Amato M., Taylor K.D., Lee J.C., Goyette P., Imielinski M., Latiano A. (2011). Meta-analysis identifies 29 additional ulcerative colitis risk loci, increasing the number of confirmed associations to 47. Nat. Genet..

[73] Franke A., McGovern D.P.B., Barrett J.C., Wang K., Radford-Smith G.L., Ahmad T., Lees C.W., Balschun T., Lee J., Roberts R. (2010). Genome-wide meta-analysis increases to 71 the number of confirmed Crohn’s disease susceptibility loci. Nat. Genet..

[74] Hugot J.-P., Chamaillard M., Zouali H., Lesage S., Cézard J.-P., Belaiche J., Almer S., Tysk C., O’Morain C.A., Gassull M. (2001). Association of NOD2 leucine-rich repeat variants with susceptibility to Crohn’s disease. Nature.

[75] Buhner S., Buning C., Genschel J., Kling K., Herrmann D., Dignass A., Kuechler I., Krueger S., Schmidt H.H.-J., Lochs H. (2006). Genetic basis for increased intestinal permeability in families with Crohn’s disease: Role of CARD15 3020insC mutation?. Gut.

[76] Gassler N., Rohr C., Schneider A., Kartenbeck J., Bach A., Obermüller N., Otto H.F., Autschbach F. (2001). Inflammatory bowel disease is associated with changes of enterocytic junctions. Am. J. Physiol. Gastrointest. Liver Physiol..

[77] Ma T.Y., Iwamoto G.K., Hoa N.T., Akotia V., Pedram A., Boivin M.A., Said H.M. (2004). TNF-α-induced increase in intestinal epithelial tight junction permeability requires NF-κB activation. Am. J. Physiol. Gastrointest. Liver Physiol..

[78] Mankertz J., Amasheh M., Krug S.M., Fromm A., Hillenbrand B., Tavalali S., Fromm M., Schulzke J.D. (2009). TNFα up-regulates claudin-2 expression in epithelial HT-29/B6 cells via phosphatidylinositol-3-kinase signaling. Cell Tissue Res..

[79] Heller F., Florian P., Bojarski C., Richter J., Christ M., Hillenbrand B., Mankertz J., Gitter A.H., Bürgel N., Fromm M. (2005). Interleukin-13 Is the Key Effector Th2 Cytokine in Ulcerative Colitis That Affects Epithelial Tight Junctions, Apoptosis, and Cell Restitution. Gastroenterology.

[80] Blair S.A., Kane S.V., Clayburgh D., Turner J.R. (2006). Epithelial myosin light chain kinase expression and activity are upregulated in inflammatory bowel disease. Lab. Investig..

[81] Zolotarevsky Y., Hecht G., Koutsouris A., Gonzalez D.E., Quan C., Tom J., Mrsny R.J., Turner J.R. (2002). A membrane-permeant peptide that inhibits MLC kinase restores barrier function in in vitro models of intestinal disease. Gastroenterology.

[82] Fischer A., Gluth M., Pape U.-F., Wiedenmann B., Theuring F., Baumgart D.C. (2013). Adalimumab prevents barrier dysfunction and antagonizes distinct effects of TNF-α on tight junction proteins and signaling pathways in intestinal epithelial cells. Am. J. Physiol. Gastrointest. Liver Physiol..

[83] Prasad S., Mingrino R., Kaukinen K., Hayes K.L., Powell R.M., Macdonald T.T., Collins J. (2005). Inflammatory processes have differential effects on claudins 2, 3 and 4 in colonic epithelial cells. Lab. Investig..

[84] Kruidenier L., Kuiper I., Lamers C.B.H.W., Verspaget H.W. (2003). Intestinal oxidative damage in inflammatory bowel disease: Semi-quantification, localization, and association with mucosal antioxidants. J. Pathol..

[85] Strus M., Gosiewski T., Fyderek K., Wedrychowicz A., Kowalska-Duplaga K., Kochan P., Adamski P., Heczko P.B. (2009). A role of hydrogen peroxide producing commensal bacteria present in colon of adolescents with inflammatory bowel disease in perpetuation of the inflammatory process. J. Physiol. Pharmacol..

[86] Morgan X.C., Tickle T., Sokol H., Gevers D., Devaney K.L., Ward D.V., Reyes J.A., Shah S.A., Leleiko N., Snapper S.B. (2012). Dysfunction of the intestinal microbiome in inflammatory bowel disease and treatment. Genome Biol..

[87] Fraumeni J.F., Hoover R.N., Devasa S.S., Kinlen J.L., Devita V.T., Hellmann S., Rosenberg S.A. (1989). Epidemiology of Cancer. Cancer: Principles and Practice of Oncology.

[88] Saito Y., Desai R.R., Muthuswamy S.K. (2018). Reinterpreting polarity and cancer: The changing landscape from tumor suppression to tumor promotion. Biochim. Biophys. Acta Rev. Cancer.

[89] Hinck L., Näthke I. (2014). Changes in cell and tissue organization in cancer of the breast and colon. Curr. Opin. Cell Biol..

[90] Martínez-Palomo A. (1970). Ultrastructural modifications of intercellular junctions between tumor cells. In Vitro.

[91] Alroy J. (1979). Tight junctions adjacent to tumor stromal interface in human invasive transitional cell carcinomas. Virchows Arch. B Cell Pathol. Incl. Mol. Pathol..

[92] Polak-Charcon S., Shoham J., Ben-Shaul Y. (1980). Tight Junctions in Epithelial Cells of Human Fetal Hindgut, Normal Colon, and Colon Adenocarcinoma. J. Natl. Cancer Inst..

[93] Robenek H., Schöpper C., Fasske E., Fetting R., Themann H. (1981). Structure and function of the junctional complement of spontaneous and transplanted murine mammary carcinomas. J. Submicrosc. Cytol..

[94] Swift J.G., Mukherjee T.M., Rowland R. (1983). Intercellular junctions in hepatocellular carcinoma. J. Submicrosc. Cytol..

[95] Zhong Y., Enomoto K., Tobioka H., Konishi Y., Satoh M., Mori M. (1994). Sequential Decrease in Tight Junctions as Revealed by 7H6 Tight Junction-associated Protein during Rat Hepatocarcinogenesis. Jpn. J. Cancer Res..

[96] Soler A.P., Miller R., Laughlin K.V., Carp N.Z., Klurfeld D., Mullin J. (1999). Increased tight junctional permeability is associated with the development of colon cancer. Carcinogenesis.

[97] Jesaitis L.A., Goodenough D.A. (1994). Molecular characterization and tissue distribution of ZO-2, a tight junction protein homologous to ZO-1 and the Drosophila discs-large tumor suppressor protein. J. Cell Biol..

[98] Willott E., Balda M., Fanning A.S., Jameson B., Van Itallie C., Anderson J. (1993). The tight junction protein ZO-1 is homologous to the Drosophila discs-large tumor suppressor protein of septate junctions. Proc. Natl. Acad. Sci. USA.

[99] Nakamura T., Blechman J., Tada S., Rozovskaia T., Itoyama T., Bullrich F., Mazo A., Croce C.M., Geiger B., Canaani E. (2000). huASH1 protein, a putative transcription factor encoded by a human homologue of the Drosophila ash1 gene, localizes to both nuclei and cell-cell tight junctions. Proc. Natl. Acad. Sci. USA.

[100] Kage H., Flodby P., Zhou B., Borok Z. (2019). Dichotomous roles of claudins as tumor promoters or suppressors: Lessons from knockout mice. Cell Mol Life Sci..

[101] Boutwell R.K., Sivak A. (1974). The Function and Mechanism of Promoters of Carcinogenesis. CRC Crit. Rev. Toxicol..

[102] Mullin J., Soler A., Laughlin K., Kampherstein J., Russo L., Saladik D., George K., Shurina R., O’Brien T. (1996). Chronic Exposure of LLC-PK1Epithelia to the Phorbol Ester TPA Produces Polyp-like Foci with Leaky Tight Junctions and Altered Protein Kinase C-α Expression and Localization. Exp. Cell Res..

[103] Dodane V., Kachar B. (1996). Identification of Isoforms of G Proteins and PKC that Colocalize with Tight Junctions. J. Membr. Biol..

[104] Clarke H., Marano C.W., Soler A.P., Mullin J.M. (2000). Modification of tight junction function by protein kinase C isoforms. Adv. Drug Deliv. Rev..

[105] Buse P., Woo P.L., Alexander D.B., Cha H.H., Reza A., Sirota N.D., Firestone G.L. (1995). Transforming Growth Factor-α Abrogates Glucocorticoid-stimulated Tight Junction Formation and Growth Suppression in Rat Mammary Epithelial Tumor Cells. J. Biol. Chem..

[106] Muthuswamy S., Li D., Lelievre S., Bissell M.J., Brugge J.S. (2001). ErbB2, but not ErbB1, reinitiates proliferation and induces luminal repopulation in epithelial acini. Nat. Cell Biol..

[107] Mullin J.M. (2004). Epithelial Barriers, Compartmentation, and Cancer. Sci. STKE.

[108] Pozzi A., Zent R. (2010). ZO-1 and ZONAB Interact to Regulate Proximal Tubular Cell Differentiation. J. Am. Soc. Nephrol..

[109] Balda M.S., Garrett M.D., Matter K. (2003). The ZO-1–associated Y-box factor ZONAB regulates epithelial cell proliferation and cell density. J. Cell Biol..

[110] Kohno T., Konno T., Kojima T. (2019). Role of Tricellular Tight Junction Protein Lipolysis-Stimulated Lipoprotein Receptor (LSR) in Cancer Cells. Int. J. Mol. Sci..

[111] Bhat A.A., Syed N., Therachiyil L., Nisar S., Hashem S., Macha M., Yadav S.K., Krishnankutty R., Muralitharan S., Al-Naemi H. (2020). Claudin-1, A Double-Edged Sword in Cancer. Int. J. Mol. Sci..

[112] Runkle E.A., Mu D. (2013). Tight junction proteins: From barrier to tumorigenesis. Cancer Lett..

[113] González-Mariscal L., Lechuga S., Garay E. (2007). Role of tight junctions in cell proliferation and cancer. Prog. Histochem. Cytochem..

[114] Kyuno D., Takasawa A., Kikuchi S., Takemasa I., Osanai M., Kojima T. (2021). Role of tight junctions in the epithelial-to-mesenchymal transition of cancer cells. Biochim. Biophys. Acta Biomembr..

[115] Venugopal S., Anwer S., Szászi K. (2019). Claudin-2: Roles beyond Permeability Functions. Int. J. Mol. Sci..

[116] Cardoso-Silva D., Delbue D., Itzlinger A., Moerkens R., Withoff S., Branchi F., Schumann M. (2019). Intestinal Barrier Function in Gluten-Related Disorders. Nutrients.

[117] Asri N., Rostami-Nejad M., Rezaei-Tavirani M., Razzaghi M., Asadzadeh-Aghdaei H., Zali M.R. (2020). Novel Therapeutic Strategies for Celiac Disease. Middle East J. Dig. Dis..

[118] Valitutti F., Fasano A. (2019). Breaking Down Barriers: How Understanding Celiac Disease Pathogenesis Informed the Development of Novel Treatments. Dig. Dis. Sci..

[119] Vancamelbeke M., Vermeire S. (2017). The intestinal barrier: A fundamental role in health and disease. Expert Rev. Gastroenterol. Hepatol..

[120] Schumann M., Siegmund B., Schulzke J.D., Fromm M. (2017). Celiac Disease: Role of the Epithelial Barrier. Cell. Mol. Gastroenterol. Hepatol..

[121] Barmeyer C., Fromm M., Schulzke J.-D. (2017). Active and passive involvement of claudins in the pathophysiology of intestinal inflammatory diseases. Pflügers Arch. Eur. J. Physiol..

[122] Barmeyer C., Schulzke J.D., Fromm M. (2015). Claudin-related intestinal diseases. Semin. Cell Dev. Biol..

[123] Schumann M., Kamel S., Pahlitzsch M.-L., Lebenheim L., May C., Krauss M., Hummel M., Daum S., Fromm M., Schulzke J.-D. (2012). Defective tight junctions in refractory celiac disease. Ann. N. Y. Acad. Sci..

[124] Vogelsang H., Schwarzenhofer M., Oberhuber G. (1998). Changes in Gastrointestinal Permeability in Celiac Disease. Dig. Dis..

[125] Kumar V., Gutierrez-Achury J., Kanduri K., Almeida R., Hrdlickova B., Zhernakova D.V., Westra H.-J., Karjalainen J., Ricaño-Ponce I., Li Y. (2015). Systematic annotation of celiac disease loci refines pathological pathways and suggests a genetic explanation for increased interferon-gamma levels. Hum. Mol. Genet..

[126] Almeida R., Ricaño-Ponce I., Kumar V., Deelen P., Szperl A., Trynka G., Gutierrez-Achury J., Kanterakis A., Westra H.-J., Franke L. (2013). Fine mapping of the celiac disease-associated LPP locus reveals a potential functional variant. Hum. Mol. Genet..

[127] Jauregi-Miguel A., Fernandez-Jimenez N., Irastorza I., Plaza-Izurieta L., Vitoria J.C., Bilbao J.R. (2014). Alteration of Tight Junction Gene Expression in Celiac Disease. J. Pediatr. Gastroenterol. Nutr..

[128] Kohl D., Ashkenazi A., Ben-Shaul Y., Bacher A. (1987). Tight junctions of jejunal surface and crypt cells in celiac disease: A freeze-fracture study. J. Pediatr. Gastroenterol. Nutr..

[129] Schulzke J.-D., Bentzel C.J., Schulzke I., Riecken E.-O., Fromm M. (1998). Epithelial Tight Junction Structure in the Jejunum of Children with Acute and Treated Celiac Sprue. Pediatr. Res..

[130] Reims A., Strandvik B., Sjövall H. (2006). Epithelial Electrical Resistance as a Measure of Permeability Changes in Pediatric Duodenal Biopsies. J. Pediatr. Gastroenterol. Nutr..

[131] Mishra A., Prakash S., Sreenivas V., Das T.K., Ahuja V., Gupta S.D., Makharia G.K. (2016). Structural and Functional Changes in the Tight Junctions of Asymptomatic and Serology-negative First-degree Relatives of Patients with Celiac Disease. J. Clin. Gastroenterol..

[132] Sowińska A., Morsy Y., Czarnowska E., Oralewska B., Konopka E., Woynarowski M., Szymańska S., Ejmont M., Scharl M., Bierła J.B. (2020). Transcriptional and Ultrastructural Analyses Suggest Novel Insights into Epithelial Barrier Impairment in Celiac Disease. Cells.

[133] Al-Sadi R., Khatib K., Guo S., Ye D., Youssef M., Ma T. (2011). Occludin regulates macromolecule flux across the intestinal epithelial tight junction barrier. Am. J. Physiol. Gastrointest. Liver Physiol..

[134] Ciccocioppo R., Finamore A., Ara C., Di Sabatino A., Mengheri E., Corazza G.R. (2006). Altered Expression, Localization, and Phosphorylation of Epithelial Junctional Proteins in Celiac Disease. Am. J. Clin. Pathol..

[135] Pizzuti D., Bortolami M., Mazzon E., Buda A., Guariso G., D’Odorico A., Chiarelli S., D’Incà R., De Lazzari F., Martines D. (2004). Transcriptional downregulation of tight junction protein ZO-1 in active coeliac disease is reversed after a gluten-free diet. Dig. Liver Dis..

[136] Goswami P., Das P., Verma A.K., Prakash S., Das T.K., Nag T.C., Ahuja V., Gupta S.D., Makharia G.K. (2014). Are alterations of tight junctions at molecular and ultrastructural level different in duodenal biopsies of patients with celiac disease and Crohn’s disease?. Virchows Arch..

[137] Szakál D.N., Győrffy H., Arato A., Cseh A., Molnár K., Papp M., Dezsőfi A., Veres G. (2010). Mucosal expression of claudins 2, 3 and 4 in proximal and distal part of duodenum in children with coeliac disease. Virchows Arch..

[138] Hollon J., Puppa E.L., Greenwald B., Goldberg E., Guerrerio A., Fasano A. (2015). Effect of Gliadin on Permeability of Intestinal Biopsy Explants from Celiac Disease Patients and Patients with Non-Celiac Gluten Sensitivity. Nutrients.

[139] Lammers K.M., Lu R., Brownley J., Lu B., Gerard C., Thomas K., Rallabhandi P., Shea-Donohue T., Tamiz A., Alkan S. (2008). Gliadin Induces an Increase in Intestinal Permeability and Zonulin Release by Binding to the Chemokine Receptor CXCR3. Gastroenterology.

[140] Sander G.R., Cummins A.G., Powell B.C. (2005). Rapid disruption of intestinal barrier function by gliadin involves altered expression of apical junctional proteins. FEBS Lett..

[141] Menard S., Lebreton C., Schumann M., Matysiak-Budnik T., Dugave C., Bouhnik Y., Malamut G., Cellier C., Allez M., Crenn P. (2012). Paracellular versus Transcellular Intestinal Permeability to Gliadin Peptides in Active Celiac Disease. Am. J. Pathol..

[142] Fasano A., Not T., Wang W., Uzzau S., Berti I., Tommasini A., Goldblum S.E. (2000). Zonulin, a newly discovered modulator of intestinal permeability, and its expression in coeliac disease. Lancet.

[143] Troisi J., Venutolo G., Terracciano C., Carri M.D., Di Micco S., Landolfi A., Fasano A. (2021). The Therapeutic use of the Zonulin Inhibitor AT-1001 (Larazotide) for a Variety of Acute and Chronic Inflammatory Diseases. Curr. Med. Chem..

[144] Nielsen H.L., Nielsen H., Ejlertsen T., Engberg J., Günzel D., Zeitz M., Hering N.A., Fromm M., Schulzke J.-D., Bücker R. (2011). Oral and Fecal Campylobacter concisus Strains Perturb Barrier Function by Apoptosis Induction in HT-29/B6 Intestinal Epithelial Cells. PLoS ONE.

[145] Bücker R., Krug S., Fromm A., Nielsen H.L., Fromm M., Nielsen H., Schulzke J.-D. (2017). Campylobacter fetusimpairs barrier function in HT-29/B6 cells through focal tight junction alterations and leaks. Ann. N. Y. Acad. Sci..

[146] Bücker R., Krug S., Moos V., Bojarski C., Schweiger M.R., Kerick M., Fromm A., Janßen S., Fromm M., Hering N.A. (2017). *Campylobacter jejuni* impairs sodium transport and epithelial barrier function via cytokine release in human colon. Mucosal Immunol..

[147] de Sá F.D.L., Backert S., Nattramilarasu P.K., Mousavi S., Sandle G.I., Bereswill S., Heimesaat M.M., Schulzke J.-D., Bücker R. (2021). Vitamin D Reverses Disruption of Gut Epithelial Barrier Function Caused by *Campylobacter jejuni*. Int. J. Mol. Sci..

[148] Bücker R., Troeger H., Kleer J., Fromm M., Schulzke J.-D. (2009). Arcobacter butzleriInduces Barrier Dysfunction in Intestinal HT-29/B6 Cells. J. Infect. Dis..

[149] Karadas G., Bücker R., Sharbati S., Schulzke J.-D., Alter T., Gölz G. (2016). Arcobacter butzleri isolates exhibit pathogenic potential in intestinal epithelial cell models. J. Appl. Microbiol..

[150] Hering N.A., Richter J.F., Krug S.M., Günzel D., Fromm A., Bohn E., Rosenthal R., Bücker R., Fromm M., Troeger H. (2010). Yersinia enterocolitica induces epithelial barrier dysfunction through regional tight junction changes in colonic HT-29/B6 cell monolayers. Lab. Investig..

[151] Bücker R., Krug S.M., Rosenthal R., Günzel D., Fromm A., Zeitz M., Chakraborty T., Fromm M., Epple H.-J., Schulzke J.-D. (2011). Aerolysin from Aeromonas hydrophila Perturbs Tight Junction Integrity and Cell Lesion Repair in Intestinal Epithelial HT-29/B6 Cells. J. Infect. Dis..

[152] Bücker R., Zakrzewski S.S., Wiegand S., Pieper R., Fromm A., Fromm M., Günzel D., Schulzke J.-D. (2020). Zinc prevents intestinal epithelial barrier dysfunction induced by alpha-hemolysin-producing Escherichia coli 536 infection in porcine colon. Vet. Microbiol..

[153] Wiegand S., Zakrzewski S.S., Eichner M., Schulz E., Günzel D., Pieper R., Rosenthal R., Barmeyer C., Bleich A., Dobrindt U. (2017). Zinc treatment is efficient against Escherichia coli α-haemolysin-induced intestinal leakage in mice. Sci. Rep..

[154] Pérez-Bosque A., Amat C., Polo J., Campbell J.M., Crenshaw J., Russell L., Moretó M. (2006). Spray-Dried Animal Plasma Prevents the Effects of *Staphylococcus aureus* Enterotoxin B on Intestinal Barrier Function in Weaned Rats. J. Nutr..

[155] Sumitomo T., Nakata M., Higashino M., Yamaguchi M., Kawabata S. (2016). Group A Streptococcus exploits human plasminogen for bacterial translocation across epithelial barrier via tricellular tight junctions. Sci. Rep..

[156] Pujol C., Eugène E., Martin L.D.S., Nassif X. (1997). Interaction of *Neisseria meningitidis* with a polarized monolayer of epithelial cells. Infect. Immun..

[157] Shrestha A., Uzal F.A., McClane B.A. (2016). The interaction of *Clostridium perfringens* enterotoxin with receptor claudins. Anaerobe.

[158] Gohari I.M., Li J., Navarro M., Uzal F., McClane B., Gohari M., Uzal L. (2019). Effects of Claudin-1 on the Action of *Clostridium perfringens* Enterotoxin in Caco-2 Cells. Toxins.

[159] Eichner M., Augustin C., Fromm A., Piontek A., Walther W., Bücker R., Fromm M., Krause G., Schulzke J.-D., Günzel D. (2017). In Colon Epithelia, *Clostridium perfringens* Enterotoxin Causes Focal Leaks by Targeting Claudins Which are Apically Accessible Due to Tight Junction Derangement. J. Infect. Dis..

[160] Vecchio A.J., Rathnayake S.S., Stroud R.M. (2021). Structural basis for *Clostridium perfringens* enterotoxin targeting of claudins at tight junctions in mammalian gut. Proc. Natl. Acad. Sci. USA.

[161] Stephens D.S., Farley M.M. (1991). Pathogenic Events during Infection of the Human Nasopharynx with *Neisseria meningitidis* and *Haemophilus influenzae*. Rev. Infect. Dis..

[162] Clarke T.B., Francella N., Huegel A., Weiser J.N. (2011). Invasive Bacterial Pathogens Exploit TLR-Mediated Downregulation of Tight Junction Components to Facilitate Translocation across the Epithelium. Cell Host Microbe.

[163] Malik Z., Roscioli E., Murphy J., Ou J., Bassiouni A., Wormald P.-J., Vreugde S. (2015). *Staphylococcus aureusimpairs* the airway epithelial barrier in vitro. Int. Forum Allergy Rhinol..

[164] Martens K., Seys S.F., Alpizar Y.A., Schrijvers R., Bullens D.M.A., Breynaert C., Lebeer S., Steelant B. (2021). *Staphylococcus aureus* enterotoxin B disrupts nasal epithelial barrier integrity. Clin. Exp. Allergy.

[165] Ohnemus U., Kohrmeyer K., Houdek P., Rohde H., Wladykowski E., Vidal S., Horstkotte M.A., Aepfelbacher M., Kirschner N., Behne M.J. (2008). Regulation of Epidermal Tight-Junctions (TJ) during Infection with Exfoliative Toxin-Negative Staphylococcus Strains. J. Investig. Dermatol..

[166] Rodríguez-Tirado C., Maisey K., Rodríguez F.E., Reyes-Cerpa S., Reyes-López F.E., Imarai M. (2012). Neisseria gonorrhoeae induced disruption of cell junction complexes in epithelial cells of the human genital tract. Microbes Infect..

[167] McLoughlin A., Rochfort K.D., McDonnell C.J., Kerrigan S.W., Cummins P.M. (2016). *Staphylococcus aureus*-mediated blood-brain barrier injury: Anin vitrohuman brain microvascular endothelial cell model. Cell. Microbiol..

[168] Liu T., Milia E., Warburton R.R., Hill N.S., Gaestel M., Kayyali U.S. (2012). Anthrax lethal toxin disrupts the endothelial permeability barrier through blocking p38 signaling. J. Cell. Physiol..

[169] McHale T.M., Garciarena C.D., Fagan R.P., Smith S.G.J., Martin-Loches I., Curley G., Fitzpatrick F., Kerrigan S.W. (2018). Inhibition of Vascular Endothelial Cell Leak Following Escherichia coli Attachment in an Experimental Model of Sepsis. Crit. Care Med..

[170] Ye P., Harty D., Commandeur Z., Hunter N. (2013). Binding of Streptococcus gordonii to oral epithelial monolayers increases paracellular barrier function. Microb. Pathog..

[171] Takai T., Ikeda S. (2011). Barrier Dysfunction Caused by Environmental Proteases in the Pathogenesis of Allergic Diseases. Allergol. Int..

[172] Al-Obaidi M.M.J., Desa M.N.M. (2018). Mechanisms of Blood Brain Barrier Disruption by Different Types of Bacteria, and Bacterial–Host Interactions Facilitate the Bacterial Pathogen Invading the Brain. Cell. Mol. Neurobiol..

[173] Lucas R., Hadizamani Y., Gonzales J., Gorshkov B., Bodmer T., Berthiaume Y., Moehrlen U., Lode H., Huwer H., Hudel M. (2020). Impact of Bacterial Toxins in the Lungs. Toxins.

[174] Freimuth P., Philipson L., Carson S.D. (2008). The coxsackievirus and adenovirus receptor. Curr. Top. Microbiol. Immunol..

[175] Torres-Flores J.M., Arias C.F. (2015). Tight Junctions Go Viral!. Viruses.

[176] Obert G., Peiffer I., Servin A.L. (2000). Rotavirus-induced structural and functional alterations in tight junctions of polarized intestinal caco-2 cell monolayers. J. Virol..

[177] Nava P., Lopez S., Arias C.F., Islas S., Gonzalez-Mariscal L. (2004). The rotavirus surface protein VP8 modulates the gate and fence function of tight junctions in epithelial cells. J. Cell Sci..

[178] Torres-Flores J.M., Silva-Ayala D., Espinoza M.A., Lopez S., Arias C.F. (2015). The tight junction protein JAM-A functions as coreceptor for rotavirus entry into MA104 cells. Virology.

[179] Tafazoli F., Zeng C.Q., Estes M.K., Magnusson K.E., Svensson L. (2001). NSP4 enterotoxin of rotavirus induces paracellular leakage in polarized epithelial cells. J. Virol..

[180] Zhao Y., Ran Z., Jiang Q., Hu N., Yu B., Zhu L., Shen L., Zhang S., Chen L., Chen H. (2019). Vitamin D Alleviates Rotavirus Infection through a Microrna-155-5p Mediated Regulation of the TBK1/IRF3 Signaling Pathway In Vivo and In Vitro. Int. J. Mol. Sci..

[181] Colgate E.R., Haque R., Dickson D.M., Carmolli M.P., Mychaleckyj J.C., Nayak U., Qadri F., Alam M., Walsh M.C., Diehl S.A. (2016). Delayed Dosing of Oral Rotavirus Vaccine Demonstrates Decreased Risk of Rotavirus Gastroenteritis Associated with Serum Zinc: A Randomized Controlled Trial. Clin. Infect. Dis..

[182] Talavera D., Castillo A.M., Dominguez M.C., Gutierrez A.E., Meza I. (2004). IL8 release, tight junction and cytoskeleton dynamic reorganization conducive to permeability increase are induced by dengue virus infection of microvascular endothelial monolayers. J. Gen. Virol..

[183] Modhiran N., Watterson D., Muller D.A., Panetta A.K., Sester D.P., Liu L., Hume D.A., Stacey K.J., Young P.R. (2015). Dengue virus NS1 protein activates cells via Toll-like receptor 4 and disrupts endothelial cell monolayer integrity. Sci. Transl. Med..

[184] Beatty P.R., Puerta-Guardo H., Killingbeck S.S., Glasner D.R., Hopkins K., Harris E. (2015). Dengue virus NS1 triggers endothelial permeability and vascular leak that is prevented by NS1 vaccination. Sci. Transl. Med..

[185] Puerta-Guardo H., Glasner D.R., Harris E. (2016). Dengue Virus NS1 Disrupts the Endothelial Glycocalyx, Leading to Hyperpermeability. PLoS Pathog..

[186] Puerta-Guardo H., Glasner D.R., Espinosa D.A., Biering S.B., Patana M., Ratnasiri K., Wang C., Beatty P.R., Harris E. (2019). Flavivirus NS1 Triggers Tissue-Specific Vascular Endothelial Dysfunction Reflecting Disease Tropism. Cell Rep..

[187] Medigeshi G.R., Hirsch A.J., Brien J.D., Uhrlaub J.L., Mason P.W., Wiley C., Nikolich-Zugich J., Nelson J.A. (2009). West nile virus capsid degradation of claudin proteins disrupts epithelial barrier function. J. Virol..

[188] Xu Z., Waeckerlin R., Urbanowski M.D., van Marle G., Hobman T.C. (2012). West Nile virus infection causes endocytosis of a specific subset of tight junction membrane proteins. PLoS ONE.

[189] Miranda J., Martin-Tapia D., Valdespino-Vazquez Y., Alarcon L., Espejel-Nunez A., Guzman-Huerta M., Muñoz-Medina J.E., Shibayama M., Chávez-Munguía B., Estrada-Gutiérrez G. (2019). Syncytiotrophoblast of Placentae from Women with Zika Virus Infection Has Altered Tight Junction Protein Expression and Increased Paracellular Permeability. Cells.

[190] Leda A., Bertrand L., Andras I.E., El-Hage N., Nair M., Toborek M. (2019). Selective Disruption of the Blood–Brain Barrier by Zika Virus. Front. Microbiol..

[191] Evans M.J., von Hahn T., Tscherne D.M., Syder A.J., Panis M., Wölk B., Hatziioannou T., McKeating J.A., Bieniasz P.D., Rice C.M. (2007). Claudin-1 is a hepatitis C virus co-receptor required for a late step in entry. Nature.

[192] Meertens L., Bertaux C., Cukierman L., Cormier E., Lavillette D., Cosset F.L., Dragic T. (2008). The tight junction proteins claudin-1, -6, and -9 are entry cofactors for hepatitis C virus. J. Virol..

[193] Benedicto I., Molina-Jiménez F., Barreiro O., Maldonado-Rodríguez A., Prieto J., Moreno-Otero R., Aldabe R., López-Cabrera M., Majano P.L. (2008). Hepatitis C virus envelope components alter localization of hepatocyte tight junction-associated proteins and promote occludin retention in the endoplasmic reticulum. Hepatology.

[194] Benedicto I., Molina-Jiménez F., Bartosch B., Cosset F.L., Lavillette D., Prieto J., Moreno-Otero R., Valenzuela-Fernández A., Aldabe R., López-Cabrera M. (2009). The tight junction-associated protein occludin is required for a postbinding step in hepatitis C virus entry and infection. J. Virol..

[195] Baktash Y., Madhav A., Coller K.E., Randall G. (2018). Single Particle Imaging of Polarized Hepatoma Organoids upon Hepatitis C Virus Infection Reveals an Ordered and Sequential Entry Process. Cell Host Microbe.

[196] Gupta S., Read S.A., Shackel N.A., Hebbard L., George J., Ahlenstiel G. (2019). The Role of Micronutrients in the Infection and Subsequent Response to Hepatitis C Virus. Cells.

[197] Long J.S., Mistry B., Haslam S.M., Barclay W.S. (2019). Host and viral determinants of influenza A virus species specificity. Nat. Rev. Microbiol..

[198] Obenauer J.C., Denson J., Mehta P.K., Su X., Mukatira S., Finkelstein D.B., Xu X., Wang J., Ma J., Fan Y. (2006). Large-scale sequence analysis of avian influenza isolates. Science.

[199] Golebiewski L., Liu H., Javier R.T., Rice A.P. (2011). The avian influenza virus NS1 ESEV PDZ binding motif associates with Dlg1 and Scribble to disrupt cellular tight junctions. J. Virol..

[200] Short K.R., Kasper J., Van Der Aa S., Andeweg A.C., Zaaraoui-Boutahar F., Goeijenbier M., Richard M., Herold S., Becker C., Scott D.P. (2016). Influenza virus damages the alveolar barrier by disrupting epithelial cell tight junctions. Eur. Respir. J..

[201] Abioye A.I., Bromage S., Fawzi W. (2021). Effect of micronutrient supplements on influenza and other respiratory tract infections among adults: A systematic review and meta-analysis. BMJ Glob. Health.

[202] Cowley T.J., Weiss S.R. (2010). Murine coronavirus neuropathogenesis: Determinants of virulence. J. NeuroVirol..

[203] Bleau C., Filliol A., Samson M., Lamontagne L. (2015). Brain Invasion by Mouse Hepatitis Virus Depends on Impairment of Tight Junctions and Beta Interferon Production in Brain Microvascular Endothelial Cells. J. Virol..

[204] Zhao S., Gao J., Zhu L., Yang Q. (2014). Transmissible gastroenteritis virus and porcine epidemic diarrhoea virus infection induces dramatic changes in the tight junctions and microfilaments of polarized IPEC-J2 cells. Virus Res..

[205] Luo X., Guo L., Zhang J., Xu Y., Gu W., Feng L., Wang Y. (2017). Tight Junction Protein Occludin Is a Porcine Epidemic Diarrhea Virus Entry Factor. J. Virol..

[206] Langel S., Paim F.C., Alhamo M.A., Lager K.M., Vlasova A.N., Saif L.J. (2019). Oral vitamin A supplementation of porcine epidemic diarrhea virus infected gilts enhances IgA and lactogenic immune protection of nursing piglets. Vet. Res..

[207] Yang J., Tian G., Chen D., Mao X., He J., Zheng P., Yu J., Luo Y., Luo J., Huang Z. (2021). 1,25-Dihydroxyvitamin D3 inhibits porcine epidemic diarrhea virus replication by regulating cell cycle resumption in IPEC-J2 porcine epithelial cells. Microb. Pathog..

[208] Zhang Q., Wu T., Li S., Meng Y., Tan Z., Wu M., Yi D., Wang L., Zhao D., Hou Y. (2021). Protective Effect of Zinc Oxide and Its Association with Neutrophil Degranulation in Piglets Infected with Porcine Epidemic Diarrhea Virus. Oxidative Med. Cell. Longev..

[209] Chai W., Zakrzewski S.S., Günzel D., Pieper R., Wang Z., Twardziok S., Janczyk P., Osterrieder N., Burwinkel M. (2014). High-dose dietary zinc oxide mitigates infection with transmissible gastroenteritis virus in piglets. BMC Vet. Res..

[210] Wei Z., Burwinkel M., Palissa C., Ephraim E., Schmidt M.F. (2012). Antiviral activity of zinc salts against transmissible gastroenteritis virus in vitro. Vet. Microbiol..

[211] Chan J.F.W., Kok K.H., Zhu Z., Chu H., To K.K.W., Yuan S., Yuen K.Y. (2020). Genomic characterization of the 2019 novel human-pathogenic coronavirus isolated from a patient with atypical pneumonia after visiting Wuhan. Emerg. Microbes Infect..

[212] Huang C., Wang Y., Li X., Ren L., Zhao J., Hu Y., Zhang L., Fan G., Xu J., Gu X. (2020). Clinical features of patients infected with 2019 novel coronavirus in Wuhan, China. Lancet.

[213] Drosten C., Günther S., Preiser W., Van Der Werf S., Brodt H.R., Becker S., Rabenau H., Panning M., Kolesnikova L., Fouchier R.A. (2003). Identification of a novel coronavirus in patients with severe acute respiratory syndrome. N. Engl. J. Med..

[214] Teoh K.T., Siu Y.L., Chan W.L., Schlüter M.A., Liu C.J., Peiris J.M., Bruzzone R., Margolis B., Nal B. (2010). The SARS coronavirus E protein interacts with PALS1 and alters tight junction formation and epithelial morphogenesis. Mol. Biol. Cell..

[215] Toto A., Ma S., Malagrinò F., Visconti L., Pagano L., Stromgaard K., Gianni S. (2020). Comparing the binding properties of peptides mimicking the Envelope protein of SARS-CoV and SARS-CoV-2 to the PDZ domain of the tight junction-associated PALS1 protein. Protein Sci..

[216] De Maio F., Cascio E.L., Babini G., Sali M., Della Longa S., Tilocca B., Roncada P., Arcovito A., Sanguinetti M., Scambia G. (2020). Improved binding of SARS-CoV-2 Envelope protein to tight junction-associated PALS1 could play a key role in COVID-19 pathogenesis. Microbes Infect..

[217] Cascio E.L., Toto A., Babini G., De Maio F., Sanguinetti M., Mordente A., Della Longa S., Arcovito A. (2021). Structural determinants driving the binding process between PDZ domain of wild type human PALS1 protein and SLiM sequences of SARS-CoV E proteins. Comput. Struct. Biotechnol. J..

[218] Shepley-McTaggart A., Sagum C.A., Oliva I., Rybakovsky E., DiGuilio K., Liang J., Bedford M.T., Cassel J., Sudol M., Mullin J.M. (2021). SARS-CoV-2 Envelope (E) Protein Interacts with PDZ-Domain-2 of Host Tight Junction Protein ZO1. PLoS ONE.

[219] Bae J.H., Choe H.J., Holick M.F., Lim S. (2022). Association of vitamin D status with COVID-19 and its severity. Rev. Endocr. Metab. Disord..

[220] Qayyum S., Mohammad T., Slominski R.M., Hassan I., Tuckey R.C., Raman C., Slominski A.T. (2021). Vitamin D and lumisterol novel metabolites can inhibit SARS-CoV-2 replication machinery enzymes. Am. J. Physiol. Metab..

[221] Shalayel M.H., Al-Mazaideh G.M., Aladaileh S.H., Al-Swailmi F.K., Al-Thiabat M.G. (2020). Vitamin D is a potential inhibitor of COVID-19: In silico molecular docking to the binding site of SARS-CoV-2 endoribonuclease Nsp15. Pak. J. Pharm. Sci..

[222] Panchariya L., Khan W.A., Kuila S., Sonkar K., Sahoo S., Ghoshal A., Kumar A., Verma D.K., Hasan A., Khan M.A. (2021). Zinc^2+^ ion inhibits SARS-CoV-2 main protease and viral replication in vitro. Chem. Commun..

[223] Sosula L. (1975). Retinal capillary junctions: Ultrastructural tight junction artefacts induced by sodium ions and membrane reduction in streptozotocin diabetes. Cell Tissue Res..

[224] Antonetti D.A., Barber A.J., Khin S., Lieth E., Tarbell J.M., Gardner T.W. (1998). Vascular permeability in experimental diabetes is associated with reduced endothelial occludin content: Vascular endothelial growth factor decreases occludin in retinal endothelial cells. Penn State Retina Research Group. Diabetes.

[225] Frey T., Antonetti D. (2011). Alterations to the Blood–Retinal Barrier in Diabetes: Cytokines and Reactive Oxygen Species. Antioxid. Redox Signal..

[226] Klaassen I., Hughes J.M., Vogels I.M., Schalkwijk C.G., Van Noorden C.J., Schlingemann R.O. (2009). Altered expression of genes related to blood–retina barrier disruption in streptozotocin-induced diabetes. Exp. Eye Res..

[227] Jiang Q.-W., Kaili D., Freeman J., Lei C.-Y., Geng B.-C., Tan T., He J.-F., Shi Z., Ma J.-J., Luo Y.-H. (2019). Diabetes inhibits corneal epithelial cell migration and tight junction formation in mice and human via increasing ROS and impairing Akt signaling. Acta Pharmacol. Sin..

[228] Banks W.A. (2020). The Blood-Brain Barrier Interface in Diabetes Mellitus: Dysfunctions, Mechanisms and Approaches to Treatment. Curr. Pharm. Des..

[229] Prasad S., Sajja R.K., Naik P., Cucullo L. (2014). Diabetes Mellitus and Blood-Brain Barrier Dysfunction: An Overview. J. Pharmacovigil..

[230] Taïlé J., Patché J., Veeren B., Gonthier M.-P. (2021). Hyperglycemic Condition Causes Pro-Inflammatory and Permeability Alterations Associated with Monocyte Recruitment and Deregulated NFκB/PPARγ Pathways on Cerebral Endothelial Cells: Evidence for Polyphenols Uptake and Protective Effect. Int. J. Mol. Sci..

[231] Yoo D.Y., Yim H.S., Jung H.Y., Nam S.M., Kim J.W., Choi J.H., Seong J.K., Yoon Y.S., Kim D.W., Hwang I.K. (2016). Chronic type 2 diabetes reduces the integrity of the blood-brain barrier by reducing tight junction proteins in the hippocampus. J. Vet. Med. Sci..

[232] Nascimento J.C., Matheus V.A., Oliveira R.B., Tada S.F.S., Collares-Buzato C.B. (2021). High-Fat Diet Induces Disruption of the Tight Junction-Mediated Paracellular Barrier in the Proximal Small Intestine Before the Onset of Type 2 Diabetes and Endotoxemia. Dig. Dis. Sci..

[233] Hawkins B.T., Lundeen T.F., Norwood K.M., Brooks H.L., Egleton R.D. (2006). Increased blood–brain barrier permeability and altered tight junctions in experimental diabetes in the rat: Contribution of hyperglycaemia and matrix metalloproteinases. Diabetologia.

[234] Genser L., Aguanno D., Soula H.A., Dong L., Trystram L., Assmann K., Salem J.-E., Vaillant J.-C., Oppert J.-M., Laugerette F. (2018). Increased jejunal permeability in human obesity is revealed by a lipid challenge and is linked to inflammation and type 2 diabetes. J. Pathol..

[235] Horton F., Wright J., Smith L., Hinton P.J., Robertson M.D. (2013). Increased intestinal permeability to oral chromium (51Cr)-EDTA in human Type 2 diabetes. Diabet. Med..

[236] Sato J., Kanazawa A., Watada H. (2017). Type 2 Diabetes and Bacteremia. Ann. Nutr. Metab..

[237] Neu J., Reverte C.M., Mackey A.D., Liboni K., Tuhacek-Tenace L.M., Hatch M., Li N., Caicedo R.A., Schatz D.A., Atkinson M. (2005). Changes in Intestinal Morphology and Permeability in the BioBreeding Rat Before the Onset of Type 1 Diabetes. J. Pediatr. Gastroenterol. Nutr..

[238] Shi Y., Qian J., Zhang F., Jia B., Liu X., Hu Y., Zhang Q., Yang Y., Sun D., Jiang L. (2019). Low molecular weight heparin (nadroparin) improves placental permeability in rats with gestational diabetes mellitus via reduction of tight junction factors. Mol. Med. Rep..

[239] Wan H., Winton H.L., Soeller C., Taylor G.W., Gruenert D.C., Thompson P.J., Cannell M.B., Stewart G.A., Garrod D.R., Robinson C. (2001). The transmembrane protein occludin of epithelial tight junctions is a functional target for serine peptidases from faecal pellets of Dermatophagoides pteronyssinus. Clin. Exp. Allergy.

[240] Wan H., Winton H.L., Soeller C., Tovey E.R., Gruenert D.C., Thompson P.J., Stewart G.A., Taylor G.W., Garrod D.R., Cannell M.B. (1999). Der p 1 facilitates transepithelial allergen delivery by disruption of tight junctions. J. Clin. Investig..

[241] Baker S.F., Yin Y., Runswick S.K., Stewart G.A., Thompson P.J., Garrod D.R., Robinson C. (2003). Peptidase allergen Der p 1 initiates apoptosis of epithelial cells independently of tight junction proteolysis. Mol. Membr. Biol..

[242] Henriquez O.A., Bs K.D.B., Hoddeson E.K., Parkos C.A., Nusrat A., Wise S.K. (2013). House dust mite allergen Der p 1 effects on sinonasal epithelial tight junctions. Int. Forum Allergy Rhinol..

[243] Steelant B., Farre R., Wawrzyniak P., Belmans J., Dekimpe E., Vanheel H., Van Gerven L., Krohn I.K., Bullens D.M.A., Ceuppens J. (2016). Impaired barrier function in patients with house dust mite–induced allergic rhinitis is accompanied by decreased occludin and zonula occludens-1 expression. J. Allergy Clin. Immunol..

[244] Wang W.-C., Tsai J.-J., Kuo C.-Y., Chen H.-M., Kao S.-H. (2011). Non-proteolytic house dust mite allergen, Der p 2, upregulated expression of tight junction molecule claudin-2 associated with Akt/GSK-3β/β-catenin signaling pathway. J. Cell. Biochem..

[245] Tulic M.K., Vivinus-Nébot M., Rekima A., Medeiros S.R., Bonnart C., Shi H., Walker A., Dainese R., Boyer J., Vergnolle N. (2016). Presence of commensal house dust mite allergen in human gastrointestinal tract: A potential contributor to intestinal barrier dysfunction. Gut.

[246] Ma S.W., Ende J.A., Alvarado R., Christensen J.M., Kalish L., Sacks R., Campbell R., Rimmer J., Harvey R. (2020). Topical Vitamin D May Modulate Human Sinonasal Mucosal Responses to House Dust Mite Antigen. Am. J. Rhinol. Allergy.

[247] Bates P.J., Farr S.J., Nicholls P.J. (1995). Effect of Cotton, Hemp, and Flax Dust Extracts on Lung Permeability in the Guinea Pig. Exp. Lung Res..

[248] Robinson C., Baker S.F., Garrod D.R. (2001). Peptidase allergens, occludin and claudins. Do their interactions facilitate the development of hypersensitivity reactions at mucosal surfaces?. Clin. Exp. Allergy.

[249] Prasad A.S. (2013). Discovery of Human Zinc Deficiency: Its Impact on Human Health and Disease. Adv. Nutr..

[250] Davidson G., Kritas S., Butler R. (2007). Stressed mucosa. Nutr. Support Infants Child. Risk.

[251] Finamore A., Massimi M., Devirgiliis L.C., Mengheri E. (2008). Zinc Deficiency Induces Membrane Barrier Damage and Increases Neutrophil Transmigration in Caco-2 Cells. J. Nutr..

[252] Ranaldi G., Ferruzza S., Canali R., Leoni G., Zalewski P.D., Sambuy Y., Perozzi G., Murgia C. (2013). Intracellular zinc is required for intestinal cell survival signals triggered by the inflammatory cytokine TNFα. J. Nutr. Biochem..

[253] Iwaya H., Kashiwaya M., Shinoki A., Lee J.-S., Hayashi K., Hara H., Ishizuka S. (2011). Marginal Zinc Deficiency Exacerbates Experimental Colitis Induced by Dextran Sulfate Sodium in Rats. J. Nutr..

[254] Guthrie G.J., Aydemir T.B., Troche C., Martin A.B., Chang S.-M., Cousins R.J. (2015). Influence of ZIP14 (slc39A14) on intestinal zinc processing and barrier function. Am. J. Physiol. Gastrointest. Liver Physiol..

[255] Zhong W., McClain C.J., Cave M., Kang Y.J., Zhou Z. (2010). The role of zinc deficiency in alcohol-induced intestinal barrier dysfunction. Am. J. Physiol. Gastrointest. Liver Physiol..

[256] Joshi P.C., Mehta A., Jabber W.S., Fan X., Guidot D.M. (2009). Zinc Deficiency Mediates Alcohol-Induced Alveolar Epithelial and Macrophage Dysfunction in Rats. Am. J. Respir. Cell Mol. Biol..

[257] Ozutemiz A.O., Aydin H.H., Isler M., Celik H.A., Batur Y. (2002). Effect of omeprazole on plasma zinc levels after oral zinc administration. Indian J. Gastroenterol..

[258] Farrell C.P., Morgan M., Rudolph D.S., Hwang A., Albert N.E., Valenzano M.C., Wang X., Mercogliano G., Mullin J.M. (2011). Proton Pump Inhibitors Interfere with Zinc Absorption and Zinc Body Stores. Gastroenterol. Res..

[259] Hara H., Konishi A., Kasai T. (2000). Contribution of the cecum and colon to zinc absorption in rats. J. Nutr..

[260] Serfaty-Lacrosniere C., Wood R.J., Voytko D., Saltzman J.R., Pedrosa M., Sepe T.E., Russell R.R. (1995). Hypochlorhydria from short-term omeprazole treatment does not inhibit intestinal absorption of calcium, phosphorus, magnesium or zinc from food in humans. J. Am. Coll. Nutr..

[261] Joshaghani H., Amiriani T., Vaghari G., Besharat S., Molana A., Badeleh M., Roshandel G. (2012). Effects of omeprazole consumption on serum levels of trace elements. J. Trace Elem. Med. Biol..

[262] Saka Y., Naruse T., Matsumoto J., Takeda Y., Onogi C., Yokoi J., Kato A., Tawada N., Noda Y., Niwa S. (2021). Low Serum Zinc Concentration Is Associated with Infection Particularly in Patients with Stage 5 Chronic Kidney Disease Medicated with Proton Pump Inhibitors. J. Ren. Nutr..

[263] Cantorna M.T., Snyder L., Arora J. (2019). Vitamin A and vitamin D regulate the microbial complexity, barrier function, and the mucosal immune responses to ensure intestinal homeostasis. Crit. Rev. Biochem. Mol. Biol..

[264] Liang D., Yang Q., Tan B., Dong X., Chi S., Liu H., Zhang S. (2020). Dietary vitamin A deficiency reduces growth performance, immune function of intestine, and alters tight junction proteins of intestine for juvenile hybrid grouper (Epinephelus fuscoguttatus ♀ × Epinephelus lanceolatus ♂). Fish Shellfish Immunol..

[265] Jiang W.-D., Zhou X.-Q., Zhang L., Liu Y., Wu P., Jiang J., Kuang S.-Y., Tang L., Tang W.-N., Zhang Y.-A. (2019). Vitamin A deficiency impairs intestinal physical barrier function of fish. Fish Shellfish Immunol..

[266] Li Y., Gao Y., Cui T., Yang T., Liu L., Li T., Chen J. (2017). Retinoic Acid Facilitates Toll-Like Receptor 4 Expression to Improve Intestinal Barrier Function through Retinoic Acid Receptor Beta. Cell. Physiol. Biochem..

[267] Movahedan A., Afsharkhamseh N., Sagha H.M., Shah J.R., Milani B.Y., Milani F.Y., Logothetis H.D., Chan C.-C., Djalilian A.R. (2013). Loss of Notch1 Disrupts the Barrier Repair in the Corneal Epithelium. PLoS ONE.

[268] Chung S.S., Choi C., Wang X., Hallock L., Wolgemuth D.J. (2009). Aberrant distribution of junctional complex components in retinoic acid receptor alpha-deficient mice. Microsc. Res. Tech..

[269] Huang H.F., Yang C.S., Meyenhofer M., Gould S., Boccabella A.V. (1988). Disruption of Sustentacular (Sertoli) Cell Tight Junctions and Regression of Spermatogenesis in Vitamin-A-Defícient Rats. Acta Anat..

[270] Rearick J.I., Jetten A.M. (1989). Effect of substratum and retinoids upon the mucosecretory differentiation of airway epithelial cells in vitro. Environ. Health Perspect..

[271] Xiao L., Cui T., Liu S., Chen B., Wang Y., Yang T., Li T., Chen J. (2019). Vitamin A supplementation improves the intestinal mucosal barrier and facilitates the expression of tight junction proteins in rats with diarrhea. Nutrition.

[272] Feng D., Chen B., Zeng B., Xiao L., Yan J., Yang T., Zhu J., Li T., Wang L., Wei H. (2021). Fecal microbiota from children with vitamin A deficiency impair colonic barrier function in germ-free mice: The possible role of alterative bile acid metabolites. Nutrition.

[273] Ismail N., Morales C.R. (1992). Effects of vitamin A deficiency on the inter?Sertoli cell tight junctions and on the germ cell population. Microsc. Res. Tech..

[274] Gorodeski G.I., Eckert R.L., Pal D., Utian W.H., Rorke E.A. (1997). Retinoids regulate tight junctional resistance of cultured human cervical cells. Am. J. Physiol..

[275] Hetta H.F., Muhammad K., El-Masry E.A., Taha A.E., Ahmed E.A., Phares C., Kader H.A., Waheed Y., Zahran A.M., Yahia R. (2021). The interplay between vitamin D and COVID-19: Protective or bystander?. Eur. Rev. Med. Pharmacol. Sci..

[276] Liu N., Sun J., Wang X., Zhang T., Zhao M., Li H. (2021). Low vitamin D status is associated with coronavirus disease 2019 outcomes: A systematic review and meta-analysis. Int. J. Infect. Dis..

[277] Sizar O., Khare S., Goyal A., Bansal P., Givler A. (2021). Vitamin D Deficiency. StatPearls [Internet].

[278] Ames B.N., Grant W.B., Willett W.C. (2021). Does the High Prevalence of Vitamin D Deficiency in African Americans Contribute to Health Disparities?. Nutrients.

[279] Liu F.H., Li S.S., Li X.X., Wang S., Li M.G., Guan L., Luan T.G., Liu Z.G., Liu Z.J., Yang P.C. (2017). Vitamin D3 induces vitamin D receptor and HDAC11 binding to relieve the promoter of the tight junction proteins. Oncotarget.

[280] Assa A., Vong L., Pinnell L.J., Avitzur N., Johnson-Henry K.C., Sherman P.M. (2014). Vitamin D Deficiency Promotes Epithelial Barrier Dysfunction and Intestinal Inflammation. J. Infect. Dis..

[281] Yang K., Zhu J., Wu J., Zhong Y., Shen X., Ms B.P., Cai W. (2021). Maternal Vitamin D Deficiency Increases Intestinal Permeability and Programs Wnt/β-Catenin Pathway in BALB/C Mice. J. Parenter. Enter. Nutr..

[282] Kellermann L., Jensen K.B., Bergenheim F., Gubatan J., Chou N.D., Moss A., Nielsen O.H. (2020). Mucosal vitamin D signaling in inflammatory bowel disease. Autoimmun. Rev..

[283] Yeung C.-Y., Chiau J.-S.C., Cheng M.-L., Chan W.-T., Jiang C.-B., Chang S.-W., Liu C.-Y., Chang C.-W., Lee H.-C. (2021). Effects of Vitamin D-Deficient Diet on Intestinal Epithelial Integrity and Zonulin Expression in a C57BL/6 Mouse Model. Front. Med..

[284] Wei X., Li X., Du J., Ge X., Sun Y., Li X., Xun Z., Liu W., Wang Z.-Y., Li Y.C. (2021). Vitamin D Deficiency Exacerbates Colonic Inflammation Due to Activation of the Local Renin–Angiotensin System in the Colon. Dig. Dis. Sci..

[285] Sayeed I., Turan N., Stein D.G., Wali B. (2019). Vitamin D deficiency increases blood-brain barrier dysfunction after ischemic stroke in male rats. Exp. Neurol..

[286] Solidoro P., Bellocchia M., Facchini F. (2016). The immunobiological and clinical role of vitamin D in obstructive lung diseases. Minerva Med..

[287] Chen H., Lu R., Zhang Y.-G., Sun J. (2018). Vitamin D Receptor Deletion Leads to the Destruction of Tight and Adherens Junctions in Lungs. Tissue Barriers.

[288] Gorman S., Buckley A.G., Ling K.-M., Berry L.J., Fear V., Stick S., Larcombe A., Kicic A., Hart P.H. (2017). Vitamin D supplementation of initially vitamin D-deficient mice diminishes lung inflammation with limited effects on pulmonary epithelial integrity. Physiol. Rep..

[289] Hambidge K.M. (1992). Zinc and diarrhea. Acta Paediatr. Suppl..

[290] Folwaczny C. (1997). Zinc and Diarrhea in Infants. J. Trace Elem. Med. Biol..

[291] Lamberti L.M., Walker C.L.F., Chan K.Y., Jian W.-Y., Black R.E. (2013). Oral Zinc Supplementation for the Treatment of Acute Diarrhea in Children: A Systematic Review and Meta-Analysis. Nutrients.

[292] Hering N.A., Schulzke J.-D. (2009). Therapeutic Options to Modulate Barrier Defects in Inflammatory Bowel Disease. Dig. Dis..

[293] Amasheh M., Andres S., Amasheh S., Fromm M., Schulzke J.-D. (2009). Barrier Effects of Nutritional Factors. Ann. N. Y. Acad. Sci..

[294] Zhou Z., Zhong W. (2017). Targeting the gut barrier for the treatment of alcoholic liver disease. Liver Res..

[295] Skrovanek S., DiGuilio K., Bailey R., Huntington W., Urbas R., Mayilvaganan B., Mercogliano G., Mullin J.M. (2014). Zinc and gastrointestinal disease. World J. Gastrointest. Pathophysiol..

[296] Valenzano M.C., DiGuilio K., Mercado J., Teter M., To J., Ferraro B., Mixson B., Manley I., Baker V., Moore B.A. (2015). Remodeling of Tight Junctions and Enhancement of Barrier Integrity of the CACO-2 Intestinal Epithelial Cell Layer by Micronutrients. PLoS ONE.

[297] Shao Y.-X., Lei Z., Wolf P.G., Gao Y., Guo Y.-M., Zhang B.-K. (2017). Zinc Supplementation, via GPR39, Upregulates PKCζ to Protect Intestinal Barrier Integrity in Caco-2 Cells Challenged by *Salmonella enterica* Serovar Typhimurium. J. Nutr..

[298] Buddington R., Wong T., Howard S. (2021). Paracellular Filtration Secretion Driven by Mechanical Force Contributes to Small Intestinal Fluid Dynamics. Med. Sci..

[299] Cohen L.Y., Sekler I., Hershfinkel M. (2014). The zinc sensing receptor, ZnR/GPR39, controls proliferation and differentiation of colonocytes and thereby tight junction formation in the colon. Cell Death Dis..

[300] Zhang B., Guo Y. (2009). Supplemental zinc reduced intestinal permeability by enhancing occludin and zonula occludens protein-1 (ZO-1) expression in weaning piglets. Br. J. Nutr..

[301] Grilli E., Tugnoli B., Vitari F., Domeneghini C., Morlacchini M., Piva A., Prandini A. (2015). Low doses of microencapsulated zinc oxide improve performance and modulate the ileum architecture, inflammatory cytokines and tight junctions expression of weaned pigs. Animals.

[302] Peng P., Deng D., Chen S., Li C., Luo J., Romeo A., Li T., Tang X., Fang R. (2020). The Effects of Dietary Porous Zinc Oxide Supplementation on Growth Performance, Inflammatory Cytokines and Tight Junction’s Gene Expression in Early-Weaned Piglets. J. Nutr. Sci. Vitaminol..

[303] Zhu C., Lv H., Chen Z., Wang L., Wu X., Chen Z., Zhang W., Liang R., Jiang Z. (2017). Dietary Zinc Oxide Modulates Antioxidant Capacity, Small Intestine Development, and Jejunal Gene Expression in Weaned Piglets. Biol. Trace Elem. Res..

[304] Wang W., Van Noten N., DeGroote J., Romeo A., Vermeir P., Michiels J. (2018). Effect of zinc oxide sources and dosages on gut microbiota and integrity of weaned piglets. J. Anim. Physiol. Anim. Nutr..

[305] Wen M., Zhao H., Liu G., Chen X., Wu B., Tian G., Cai J., Jia G. (2017). Effect of Zinc Supplementation on Growth Performance, Intestinal Development, and Intestinal Barrier-Related Gene Expression in Pekin Ducks. Biol. Trace Elem. Res..

[306] Fan P., Tan Y., Jin K., Lin C., Xia S., Han B., Zhang F., Wu L., Ma X. (2017). Supplemental lipoic acid relieves post-weaning diarrhoea by decreasing intestinal permeability in rats. J. Anim. Physiol. Anim. Nutr..

[307] Bzik V.A., Medani M., Baird A., Winter D.C., Brayden D.J. (2012). Mechanisms of action of zinc on rat intestinal epithelial electrogenic ion secretion: Insights into its antidiarrhoeal actions. J. Pharm. Pharmacol..

[308] Ranaldi G., Caprini V., Sambuy Y., Perozzi G., Murgia C. (2009). Intracellular zinc stores protect the intestinal epithelium from Ochratoxin A toxicity. Toxicol. Vitr..

[309] Roselli M., Finamore A., Garaguso I., Britti M.S., Mengheri E. (2003). Zinc Oxide Protects Cultured Enterocytes from the Damage Induced by Escherichia coli. J. Nutr..

[310] Choudhry N., Scott F., Edgar M., Sanger G., Kelly P. (2021). Reversal of Pathogen-Induced Barrier Defects in Intestinal Epithelial Cells by Contra-pathogenicity Agents. Dig. Dis. Sci..

[311] Sarkar P., Saha T., Sheikh I.A., Chakraborty S., Aoun J., Chakrabarti M.K., Rajendran V.M., Ameen N.A., Dutta S., Hoque K.M. (2019). Zinc ameliorates intestinal barrier dysfunctions in shigellosis by reinstating claudin-2 and -4 on the membranes. Am. J. Physiol. Gastrointest. Liver Physiol..

[312] Song Z.-H., Ke Y.-L., Xiao K., Jiao L.-F., Hong Q.-H., Hu C.-H. (2015). Diosmectite–zinc oxide composite improves intestinal barrier restoration and modulates TGF-β1, ERK1/2, and Akt in piglets after acetic acid challenge. J. Anim. Sci..

[313] Sturniolo G.C., Fries W., Mazzon E., Di Leo V., Barollo M., D’Inca R. (2002). Effect of zinc supplementation on intestinal permeability in experimental colitis. J. Lab. Clin. Med..

[314] Zhong W., Li Q., Sun Q., Zhang W., Zhang J., Sun X., Yin X., Zhang X., Zhou Z. (2015). Preventing Gut Leakiness and Endotoxemia Contributes to the Protective Effect of Zinc on Alcohol-Induced Steatohepatitis in Rats. J. Nutr..

[315] Rodriguez P., Darmon N., Chappuis P., Candalh C., Blaton M.A., Bouchaud C., Heyman M. (1996). Intestinal paracellular permeability during malnutrition in guinea pigs: Effect of high dietary zinc. Gut.

[316] Xie Y., Wen M., Zhao H., Liu G., Chen X., Tian G., Cai J., Jia G. (2021). Effect of zinc supplementation on growth performance, intestinal development, and intestinal barrier function in Pekin ducks with lipopolysaccharide challenge. Poult. Sci..

[317] Bortoluzzi C., Lumpkins B., Mathis G., França M., King W., Graugnard D., Dawson K., Applegate T. (2019). Zinc source modulates intestinal inflammation and intestinal integrity of broiler chickens challenged with coccidia and *Clostridium perfringens*. Poult. Sci..

[318] Davison G., Marchbank T., March D.S., Thatcher R., Playford R.J. (2016). Zinc carnosine works with bovine colostrum in truncating heavy exercise–induced increase in gut permeability in healthy volunteers. Am. J. Clin. Nutr..

[319] Ben Lagha A., Yang Y., Trivedi H.M., Masters J.G., Grenier D. (2021). A Dual Zinc plus Arginine formulation protects against tumor necrosis factor-alpha-induced barrier dysfunction and enhances cell proliferation and migration in an in vitro gingival keratinocyte model. Arch. Oral Biol..

[320] Thomas P., Converse A., Berg H. (2018). ZIP9, a novel membrane androgen receptor and zinc transporter protein. Gen. Comp. Endocrinol..

[321] Song Y., Xue Y., Liu X., Wang P., Liu L. (2008). Effects of acute exposure to aluminum on blood–brain barrier and the protection of zinc. Neurosci. Lett..

[322] Qi Z., Liang J., Pan R., Dong W., Shen J., Yang Y., Zhao Y., Shi W., Luo Y., Ji X. (2016). Zinc contributes to acute cerebral ischemia-induced blood–brain barrier disruption. Neurobiol. Dis..

[323] Wang X., Valenzano M.C., Mercado J.M., Zurbach E.P., Flounders C.J., Mullin J.M. (2014). Zinc enhancement of LLC-PK1 renal epithelial barrier function. Clin. Nutr..

[324] Carr G., Wright J.A., Simmons N.L. (2010). Epithelial Barrier Resistance is Increased by the Divalent Cation Zinc in Cultured MDCKII Epithelial Monolayers. J. Membr. Biol..

[325] Jacquillet G., Barbier O., Cougnon M., Tauc M., Namorado M.C., Martin D., Reyes J.L., Poujeol P. (2006). Zinc protects renal function during cadmium intoxication in the rat. Am. J. Physiol. Physiol..

[326] Mercado J., Valenzano M.C., Jeffers C., Sedlak J., Cugliari M.K., Papanikolaou E., Clouse J., Miao J., Wertan N.E., Mullin J.M. (2013). Enhancement of Tight Junctional Barrier Function by Micronutrients: Compound-Specific Effects on Permeability and Claudin Composition. PLoS ONE.

[327] He Y., Yuan X., Zuo H., Li X., Sun Y., Feng A. (2019). Berberine induces ZIP14 expression and modulates zinc redistribution to protect intestinal mucosal barrier during polymicrobial sepsis. Life Sci..

[328] De Medeiros P.H.Q.S., Pinto D.V., De Almeida J.Z., Rêgo J.M.C., Rodrigues F.A.P., Lima A., Ângelo M., Bolick D.T., Guerrant R.L., Oriá R.B. (2018). Modulation of Intestinal Immune and Barrier Functions by Vitamin A: Implications for Current Understanding of Malnutrition and Enteric Infections in Children. Nutrients.

[329] Abdelhamid L., Luo X.M. (2018). Retinoic Acid, Leaky Gut, and Autoimmune Diseases. Nutrients.

[330] Yamada S., Kanda Y. (2019). Retinoic acid promotes barrier functions in human iPSC-derived intestinal epithelial monolayers. J. Pharmacol. Sci..

[331] Wang P.-F., Yao D.-H., Hu Y.-Y., Li Y. (2019). Vitamin D Improves Intestinal Barrier Function in Cirrhosis Rats by Upregulating Heme Oxygenase-1 Expression. Biomol. Ther..

[332] Zuo L., Yang X., Lu M., Hu R., Zhu H., Zhang S., Zhou Q., Chen F., Gui S., Wang Y. (2016). All-Trans Retinoic Acid Inhibits Human Colorectal Cancer Cells RKO Migration via Downregulating Myosin Light Chain Kinase Expression through MAPK Signaling Pathway. Nutr. Cancer.

[333] Filteau S.M., Rollins N.C., Coutsoudis A., Sullivan K.R., Willumsen J.F., Tomkins A.M. (2001). The Effect of Antenatal Vitamin A and β-Carotene Supplementation on Gut Integrity of Infants of HIV-Infected South African Women. J. Pediatr. Gastroenterol. Nutr..

[334] Lima A.A., Soares A.M., Lima N.L., Mota R.M., Maciel B.L., Kvalsund M.P., Barrett L.J., Fitzgerald R.P., Blaner W.S., Guerrant R.L. (2010). Effects of Vitamin A Supplementation on Intestinal Barrier Function, Growth, Total Parasitic, and Specific Giardia spp Infections in Brazilian Children: A Prospective Randomized, Double-blind, Placebo-controlled Trial. J. Pediatr. Gastroenterol. Nutr..

[335] He C., Hu X., Xiao D., Wu J., Zhou S., Deng J., Xu S., Huang Y., Peng M., Yang X. (2020). Vitamin A prevents lipopolysaccharide-induced injury on tight junctions in mice. Food Sci. Nutr..

[336] Xiao S., Li Q., Hu K., He Y., Ai Q., Hu L., Yu J. (2018). Vitamin A and Retinoic Acid Exhibit Protective Effects on Necrotizing Enterocolitis by Regulating Intestinal Flora and Enhancing the Intestinal Epithelial Barrier. Arch. Med Res..

[337] Cheng J., Balbuena E., Miller B., Eroglu A. (2021). The Role of β-Carotene in Colonic Inflammation and Intestinal Barrier Integrity. Front. Nutr..

[338] Maciel A.A., Oriá R.B., Braga-Neto M.B., Braga A.B., Carvalho E.B., Lucena H.B., Brito G.A., Guerrant R.L., Lima A.A. (2007). Role of retinol in protecting epithelial cell damage induced by Clostridium difficile toxin A. Toxicon.

[339] Osanai M., Nishikiori N., Murata M., Chiba H., Kojima T., Sawada N. (2006). Cellular Retinoic Acid Bioavailability Determines Epithelial Integrity: Role of Retinoic Acid Receptor α Agonists in Colitis. Mol. Pharmacol..

[340] Molina-Jijón E., Rodríguez-Muñoz R., Namorado M.D.C., Bautista-García P., Medina-Campos O.N., Pedraza-Chaverri J., Reyes J.L. (2015). All- trans retinoic acid prevents oxidative stress-induced loss of renal tight junction proteins in type-1 diabetic model. J. Nutr. Biochem..

[341] Lochbaum R., Schilpp C., Nonnenmacher L., Frick M., Dietl P., Wittekindt O.H. (2020). Retinoic acid signalling adjusts tight junction permeability in response to air-liquid interface conditions. Cell. Signal..

[342] Hatakeyama S., Ishida K., Takeda Y. (2010). Changes in cell characteristics due to retinoic acid; specifically, a decrease in the expression of claudin-1 and increase in claudin-4 within tight junctions in stratified oral keratinocytes. J. Periodontal Res..

[343] Groeger S., Jarzina F., Windhorst A., Meyle J. (2016). Influence of retinoic acid on human gingival epithelial barriers. J. Periodontal Res..

[344] Zhou Y., Zhang D., Hu D., Liu B., Peng J., Shen L., Long C., Yu Y., Zhang Y., Liu X. (2019). Retinoic acid: A potential therapeutic agent for cryptorchidism infertility based on investigation of flutamide-induced cryptorchid rats in vivo and in vitro. Reprod. Toxicol..

[345] Zhouguang W., Zhang H., Zheng B., Ye L., Zhu S., Johnson N.R., Wang Z., Wei X., Chen D., Cao G. (2016). Retinoic Acid Induced-Autophagic Flux Inhibits ER-Stress Dependent Apoptosis and Prevents Disruption of Blood-Spinal Cord Barrier after Spinal Cord Injury. Int. J. Biol. Sci..

[346] Satoh H., Zhong Y., Isomura H., Saitoh M., Enomoto K., Sawada N., Mori M. (1996). Localization of 7H6 Tight Junction-Associated Antigen along the Cell Border of Vascular Endothelial Cells Correlates with Paracellular Barrier Function against Ions, Large Molecules, and Cancer Cells. Exp. Cell Res..

[347] Rong J., Liu S. (2011). Effect of all-trans retinoic acid on the barrier function in human retinal pigment epithelial cells. Biochem. Biophys. Res. Commun..

[348] Kubota H., Chiba H., Takakuwa Y., Osanai M., Tobioka H., Kohama G.-I., Mori M., Sawada N. (2001). Retinoid X Receptor α and Retinoic Acid Receptor γ Mediate Expression of Genes Encoding Tight-Junction Proteins and Barrier Function in F9 Cells during Visceral Endodermal Differentiation. Exp. Cell Res..

[349] Tobioka H., Sawada N., Zhong Y., Mori M. (1996). Enhanced paracellular barrier function of rat mesothelial cells partially protects against cancer cell penetration. Br. J. Cancer.

[350] Retana C., Sanchez E., Perez-Lopez A., Cruz A., Lagunas J., Cruz C., Vital S., Reyes J.L. (2015). Alterations of Intercellular Junctions in Peritoneal Mesothelial Cells from Patients Undergoing Dialysis: Effect of Retinoic Acid. Perit. Dial. Int..

[351] Telgenhoff D., Ramsay S., Hilz S., Slusarewicz P., Shroot B. (2008). Claudin 2 mRNA and Protein Are Present in Human Keratinocytes and May Be Regulated by All-trans-Retinoic Acid. Ski. Pharmacol. Physiol..

[352] Elias P.M., Friend D.S. (1976). Vitamin-A-induced mucous metaplasia. An in vitro system for modulating tight and gap junction differentiation. J. Cell Biol..

[353] Gorodeski G.I., Pal D., Rorke E.A., Eckert R.L., Burfeind P. (1998). Retinoids modulate P2Upurinergic receptor-mediated changes in transcervical paracellular permeability. Am. J. Physiol. Cell Physiol..

[354] Baltes S., Nau H., Lampen A. (2004). All-trans retinoic acid enhances differentiation and influences permeability of intestinal Caco-2 cells under serum-free conditions. Dev. Growth Differ..

[355] Gubatan J., Moss A.C. (2018). Vitamin D in inflammatory bowel disease: More than just a supplement. Curr. Opin. Gastroenterol..

[356] Noriega D.B., Savelkoul H. (2021). Vitamin D and Allergy Susceptibility during Gestation and Early Life. Nutrients.

[357] Battistini C., Ballan R., Herkenhoff M., Saad S., Sun J. (2020). Vitamin D Modulates Intestinal Microbiota in Inflammatory Bowel Diseases. Int. J. Mol. Sci..

[358] Du J., Chen Y., Shi Y., Liu T., Cao Y., Tang Y., Ge X., Nie H., Zheng C., Li Y.C. (2015). 1,25-Dihydroxyvitamin D Protects Intestinal Epithelial Barrier by Regulating the Myosin Light Chain Kinase Signaling Pathway. Inflamm. Bowel Dis..

[359] Kong J., Zhang Z., Musch M.W., Ning G., Sun J., Hart J., Bissonnette M., Li Y.C. (2008). Novel role of the vitamin D receptor in maintaining the integrity of the intestinal mucosal barrier. Am. J. Physiol. Gastrointest. Liver Physiol..

[360] Lee C., Lau E., Chusilp S., Filler R., Li B., Zhu H., Yamoto M., Pierro A. (2019). Protective effects of vitamin D against injury in intestinal epithelium. Pediatr. Surg. Int..

[361] Zhao H., Zhang H., Wu H., Li H., Liu L., Guo J., Li C., Shih D.Q., Zhang X. (2012). Protective role of 1,25(OH)2vitamin D3 in the mucosal injury and epithelial barrier disruption in DSS-induced acute colitis in mice. BMC Gastroenterol..

[362] Liu T., Shi Y., Du J., Ge X., Teng X., Liu L., Wang E., Zhao Q. (2016). Vitamin D treatment attenuates 2,4,6-trinitrobenzene sulphonic acid (TNBS)-induced colitis but not oxazolone-induced colitis. Sci. Rep..

[363] Chatterjee I., Zhang Y., Zhang J., Lu R., Xia Y., Sun J. (2021). Overexpression of Vitamin D Receptor in Intestinal Epithelia Protects Against Colitis via Upregulating Tight Junction Protein Claudin 15. J. Crohn’s Colitis.

[364] Zhang Y.-G., Wu S., Lu R., Zhou D., Zhou J., Carmeliet G., Petrof E., Claud E.C., Sun J. (2015). Tight junction CLDN2 gene is a direct target of the vitamin D receptor. Sci. Rep..

[365] Liu Y., Meng F., Wang S., Xia S., Wang R. (2021). Vitamin D_3_ mitigates lipopolysaccharide-induced oxidative stress, tight junction damage and intestinal inflammatory response in yellow catfish, Pelteobagrus fulvidraco. Comp. Biochem. Physiol. Part C Toxicol. Pharmacol..

[366] Qiu F., Zhang Z., Yang L., Li R., Ma Y. (2021). Combined effect of vitamin C and vitamin D_3_ on intestinal epithelial barrier by regulating Notch signaling pathway. Nutr. Metab..

[367] Assa A., Vong L., Pinnell L.J., Rautava J., Avitzur N., Johnson-Henry K.C., Sherman P.M. (2015). Vitamin D Deficiency Predisposes to Adherent-invasive Escherichia coli-induced Barrier Dysfunction and Experimental Colonic Injury. Inflamm. Bowel Dis..

[368] Chen S.-W., Ma Y.-Y., Zhu J., Zuo S., Zhang J.-L., Chen Z.-Y., Chen G.-W., Wang X., Pan Y.-S., Liu Y.-C. (2015). Protective effect of 1,25-dihydroxyvitamin D3 on ethanol-induced intestinal barrier injury both in vitro and in vivo. Toxicol. Lett..

[369] Dong S., Singh T.P., Wei X., Yao H., Wang H. (2018). Protective Effect of 1,25-Dihydroxy Vitamin D3 on Pepsin–Trypsin-Resistant Gliadin-Induced Tight Junction Injuries. Dig. Dis. Sci..

[370] Wang Z., Li J., Wang Y., Wang L., Yin Y., Yin L., Yang H., Yin Y. (2020). Dietary vitamin A affects growth performance, intestinal development, and functions in weaned piglets by affecting intestinal stem cells. J. Anim. Sci..

[371] Liu X.Z., You B., Zhang Y.L., Yang Z.C., Chen P., Shi Y.L., Chen Y., Chen J., Peng Y.Z. (2019). Effects of vitamin D3 on intestinal mucosal barrier of mice with severe burns. Zhonghua Shao Shang Za Zhi.

[372] Lee P., Hsieh Y., Huo T., Yang U., Lin C., Li C., Huang Y., Hou M., Lin H., Lee K. (2021). Active Vitamin D 3 Treatment Attenuated Bacterial Translocation via Improving Intestinal Barriers in Cirrhotic Rats. Mol. Nutr. Food Res..

[373] Fernandez-Robredo P., González-Zamora J., Recalde S., Bilbao-Malavé V., Bezunartea J., Hernandez M., Garcia-Layana A. (2020). Vitamin D Protects against Oxidative Stress and Inflammation in Human Retinal Cells. Antioxidants.

[374] Mohanty S., Kamolvit W., Hertting O., Brauner A. (2020). Vitamin D strengthens the bladder epithelial barrier by inducing tight junction proteins during E. coli urinary tract infection. Cell Tissue Res..

[375] Shi Y.-Y., Liu T.-J., Fu J.-H., Xu W., Wu L.-L., Hou A.-N., Xue X.-D. (2016). Vitamin D/VDR signaling attenuates lipopolysaccharide-induced acute lung injury by maintaining the integrity of the pulmonary epithelial barrier. Mol. Med. Rep..

[376] Li W., Dong H., Zhao H., Song J., Tang H., Yao L., Liu L., Tong W., Zou M., Zou F. (2015). 1,25-Dihydroxyvitamin D3 prevents toluene diisocyanate-induced airway epithelial barrier disruption. Int. J. Mol. Med..

[377] Won S., Sayeed I., Peterson B.L., Wali B., Kahn J.S., Stein D.G. (2015). Vitamin D Prevents Hypoxia/Reoxygenation-Induced Blood-Brain Barrier Disruption via Vitamin D Receptor-Mediated NF-kB Signaling Pathways. PLoS ONE.

[378] Boguniewicz M., Leung D.Y.M. (2011). Atopic dermatitis: A disease of altered skin barrier and immune dysregulation. Immunol. Rev..

[379] Tudpor K., Teerapornpuntakit J., Jantarajit W., Krishnamra N., Charoenphandhu N. (2008). 1,25-Dihydroxyvitamin D3 Rapidly Stimulates the Solvent Drag–Induced Paracellular Calcium Transport in the Duodenum of Female Rats. J. Physiol. Sci..

[380] Fujita H., Sugimoto K., Inatomi S., Maeda T., Osanai M., Uchiyama Y., Yamamoto Y., Wada T., Kojima T., Yokozaki H. (2008). Tight Junction Proteins Claudin-2 and -12 Are Critical for Vitamin D-dependent Ca2+ Absorption between Enterocytes. Mol. Biol. Cell.

[381] Chirayath M.V., Gajdzik L., Hulla W., Graf J., Cross H.S., Peterlik M. (1998). Vitamin D increases tight-junction conductance and paracellular Ca2+ transport in Caco-2 cell cultures. Am. J. Physiol. Gastrointest. Liver Physiol..

[382] Kladnitsky O., Rozenfeld J., Efrati E., Zelikovic I., Azulay-Debby H. (2014). The claudin-16 channel gene is transcriptionally inhibited by 1,25-dihydroxyvitamin D. Exp. Physiol..

[383] Camilleri M. (2021). What is the leaky gut? Clinical considerations in humans. Curr. Opin. Clin. Nutr. Metab. Care.

[384] DiGuilio K.M., Rybakovsky E., Valenzano M.C., Lander J., Manganiello A., Leroy K., Bensinger A., Chen W., Chen V., Majumdar S. (2021). S1354 Modification of the Tight Junctional Barrier in Human Duodenal Mucosal by Oral Zinc Administration in a Patient-Based Study: RNA-Seq and Western Immunoblot Analyses. Am. J. Gastroenterol..

[385] Sturniolo G.C., Di Leo V., Ferronato A., D’Odorico A., D’Incà R. (2001). Zinc Supplementation Tightens “Leaky Gut” in Crohn’s Disease. Inflamm. Bowel Dis..

[386] Roy S.K., Behrens R.H., Haider R., Akramuzzaman S.M., Mahalanabis D., Wahed M.A., Tomkins A.M. (1992). Impact of Zinc Supplementation on Intestinal Permeability in Bangladeshi Children with Acute Diarrhoea and Persistent Diarrhoea Syndrome. J. Pediatr. Gastroenterol. Nutr..

[387] Alam A.N., Sarker S.A., Wahed M.A., Khatun M., Rahaman M.M. (1994). Enteric protein loss and intestinal permeability changes in children during acute shigellosis and after recovery: Effect of zinc supplementation. Gut.

[388] Ryan K.N., Stephenson K.B., Trehan I., Shulman R.J., Thakwalakwa C., Murray E., Maleta K., Manary M.J. (2014). Zinc or Albendazole Attenuates the Progression of Environmental Enteropathy: A Randomized Controlled Trial. Clin. Gastroenterol. Hepatol..

[389] Tran C.D., Hawkes J., Graham R.D., Kitchen J.L., Symonds E., Davidson G.P., Butler R.N. (2015). Zinc-Fortified Oral Rehydration Solution Improved Intestinal Permeability and Small Intestinal Mucosal Recovery. Clin. Pediatr..

[390] Tsai Y.L., Ko W.-S., Hsiao J.-L., Pan H.-H., Chiou Y.-L. (2016). Zinc sulfate improved the unbalanced T cell profiles in Der p-allergic asthma: An ex vivo study. Clin. Respir. J..

[391] Stio M., Retico L., Annese V., Bonanomi A.G. (2016). Vitamin D regulates the tight-junction protein expression in active ulcerative colitis. Scand. J. Gastroenterol..

[392] Domazetovic V., Iantomasi T., Bonanomi A.G., Stio M. (2020). Vitamin D regulates claudin-2 and claudin-4 expression in active ulcerative colitis by p-Stat-6 and Smad-7 signaling. Int. J. Color. Dis..

[393] Yang Y., Cui X., Li J., Wang H., Li Y., Chen Y., Zhang H. (2021). Clinical evaluation of vitamin D status and its relationship with disease activity and changes of intestinal immune function in patients with Crohn’s disease in the Chinese population. Scand. J. Gastroenterol..

[394] Meckel K., Li Y.C., Lim J., Kocherginsky M., Weber C., Almoghrabi A., Chen X., Kaboff A., Sadiq F., Hanauer S.B. (2016). Serum 25-hydroxyvitamin D concentration is inversely associated with mucosal inflammation in patients with ulcerative colitis. Am. J. Clin. Nutr..

[395] Dancer R.C.A., Parekh D., Lax S., D’Souza V., Zheng S., Bassford C.R., Park D., Bartis D.G., Mahida R., Turner A.M. (2015). Vitamin D deficiency contributes directly to the acute respiratory distress syndrome (ARDS). Thorax.

[396] Thurnham D.I., Northrop-Clewes C.A., McCullough F.S.W., Das B.S., Lunn P.G. (2000). Innate Immunity, Gut Integrity, and Vitamin A in Gambian and Indian Infants. J. Infect. Dis..

[397] Mueller C.M. (2017). The ASPEN Adult Nutrition Support Core Curriculum.

[398] Vanek V.W., Borum P., Buchman A., Fessler T.A., Howard L., Jeejeebhoy K., Kochevar M., Shenkin A., Valentine C.J., Novel Nutrient Task Force (2012). A.S.P.E.N. position paper: Recommendations for changes in commercially available parenteral multivitamin and multi-trace element products. Nutr. Clin. Pract..

[399] Institute of Medicine (US) Panel on Micronutrients (2001). Dietary Reference Intakes for Vitamin A, Vitamin K, Arsenic, Boron, Chromium, Copper, Iodine, Iron, Manganese, Molybdenum, Nickel, Silicon, Vanadium, and Zinc.

[400] Murphy S.P., Yates A.A., Atkinson S.A., Barr S.I., Dwyer J. (2016). History of Nutrition: The Long Road Leading to the Dietary Reference Intakes for the United States and Canada. Adv. Nutr..

[401] Vanek V.W., Borum P., Buchman A., Fessler T., Howard L., Shenkin A., Valentine C.J., Novel Nutrient Task Force, Parenteral Vitamin and Trace Element Working Group, American Society for Parenteral and Enteral Nutrition (ASPEN) (2015). A Call to Action to Bring Safer Parenteral Micronutrient Products to the U.S. Market. Nutr. Clin. Pract..

[402] American Society for Parenteral and Enteral Nutrition (2019). Appropriate Dosing for Parenteral Nutrition: ASPEN Recommendations. https://www.nutritioncare.org/uploadedFiles/Documents/Guidelines_and_Clinical_Resources/.

